# Evidence for correlations between BMI-associated SNPs and circRNAs

**DOI:** 10.1038/s41598-022-16495-7

**Published:** 2022-07-25

**Authors:** Luisa Sophie Rajcsanyi, Inga Diebels, Lydia Pastoors, Deniz Kanber, Triinu Peters, Anna-Lena Volckmar, Yiran Zheng, Martin Grosse, Christoph Dieterich, Johannes Hebebrand, Frank J. Kaiser, Bernhard Horsthemke, Anke Hinney

**Affiliations:** 1 Department of Child and Adolescent Psychiatry, Psychosomatics and Psychotherapy, University Hospital Essen, University of Duisburg-Essen, Essen, Germany; 2 Center for Translational Neuro- and Behavioural Sciences, University Hospital Essen, Essen, Germany; 3 Institute of Human Genetics, University Hospital Essen, Essen, Germany; 4 Institute of Pathology, University Hospital Heidelberg, Heidelberg, Germany; 5 Department of Internal Medicine III, University Hospital Heidelberg, Heidelberg, Germany; 6 German Center for Cardiovascular Research (DZHK), Partner site Heidelberg/Mannheim, Heidelberg, Germany

**Keywords:** Gene regulation, Medical genetics, Mutation

## Abstract

Circular RNAs (circRNAs) are regulators of processes like adipogenesis. Their expression can be modulated by SNPs. We analysed links between BMI-associated SNPs and circRNAs. First, we detected an enrichment of BMI-associated SNPs on circRNA genomic loci in comparison to non-significant variants. Analysis of sex-stratified GWAS data revealed that circRNA genomic loci encompassed more genome-wide significant BMI-SNPs in females than in males. To explore whether the enrichment is restricted to BMI, we investigated nine additional GWAS studies. We showed an enrichment of trait-associated SNPs in circRNAs for four analysed phenotypes (body height, chronic kidney disease, anorexia nervosa and autism spectrum disorder). To analyse the influence of BMI-affecting SNPs on circRNA levels in vitro, we examined rs4752856 located on hsa_circ_0022025. The analysis of heterozygous individuals revealed an increased level of circRNA derived from the BMI-increasing SNP allele. We conclude that genetic variation may affect the BMI partly through circRNAs.

## Introduction

The prevalence of obesity is increasing globally^1–3^. Due to an elevated body mass index (BMI), the risk to develop comorbidities, like cardiovascular diseases, diabetes, cancers, and Alzheimer’s disease, increases concomitantly, causing a greater risk for a premature death^3–5^. Genetic variants associated with BMI have been identified in genome-wide association studies (GWAS)^4,6–8^. The most recent meta-analysis of BMI GWAS detected 941 genetic loci associated with the BMI^9^. The functional characterisation of these single nucleotide polymorphisms (SNPs) remains challenging, as the majority of GWAS-identified signals reside within non-coding regions and are thus located in enhancers or sequences harbouring non-coding RNAs (ncRNAs), such as circular RNAs (circRNAs)^6,10,11^.

CircRNAs derive from a backsplicing process linking a downstream splice donor to an upstream splice acceptor forming covalently closed loops^12–14^. These circular transcripts are expressed in a cell-type-, tissue-, and developmental stage-specific manner, exhibiting the highest enrichment in the brain and during neurogenesis^12,14–17^. Previous studies linked circRNAs to various diseases and cellular processes, such as the preadipocyte differentiation, known as adipogenesis^18–21^. A knockdown of circSAMD4A, for instance, inhibited the adipogenesis and eventually led to a reversion of the murine weight gain when fed a high-fat diet. In humans, overexpression of circSAMD4A was associated with an increased BMI^19^. Dynamic regulation of circRNAs during adipogenesis and in obesity provided insights in the emerging regulatory roles of these transcripts. Two circRNAs, circTshz2-1 and circArhgap5-2, were found to be substantial regulators of the preadipocyte maturation as depletions of the circRNAs inhibited the respective mechanism^18^.

Evidence has emerged that genetic variations can affect circRNAs. Accordingly, the level of circRNAs is influenced in a genotype-specific manner^22,23^. For example, carriers of the C-allele of a multiple-sclerosis (MS)-associated SNP showed increased levels of a *STAT3*-derived circRNA^22^. Additionally, SNPs located within the flanking sites of circRNAs showed higher correlations with the respective circular transcript than SNPs located within the circRNA sequence^24^.

To date, there is a lack of studies exploring GWAS-identified SNPs and their impact on circRNAs, particularly in the context of BMI. Given that circRNAs are highly expressed in the brain^16^ and energy homeostasis is associated with the hypothalamus^25^, we speculate there may be links between SNPs that are associated with BMI alterations and circRNAs. Consequently, the present study aimed to investigate interactions of genome-wide significant SNPs (P < 5 × 10^−8^) identified in the most recent meta-analysis of BMI GWAS^9^ with circRNAs. We initially examined whether these significant SNPs are more frequently located on circRNA genomic loci compared to non-significant variants. Investigation of additional GWAS studies pertaining to psychiatric, neurological, anthropometric, and peripheral traits ensued. Subsequently, we selected one genome-wide significant SNP (rs4752856) for BMI, which is located on a circRNA derived from an obesity-associated gene (*MTCH2*; hsa_circ_0022025), and assessed the relative levels of the circRNA derived from the two alleles in vitro.

## Results

### Enrichment of genome-wide significant BMI-SNPs

Initially, we investigated if the proportion of genome-wide significant SNPs (P < 5 × 10^−8^) on circRNA genomic loci deviates from the ratio of non-significant SNPs (P ≥ 5 × 10^−8^) located within these regions. Therefore, we used data from the most recent BMI GWAS meta-analysis^9^ (see Table 1 and Supplementary Table S2) and four publicly available circRNA databases (circAtlas v2.0, circBase, CIRCpediaV2 and circVAR; see Supplementary Table S1). Due to different reference genome versions between circRNA and the majority of SNP data (see Supplementary Table S1 and S2), we subjected the SNP data to a genome version lift over to ensure compatibility with all circRNA databases. Notably, due to ambiguous mappings, over 10,000 SNPs had to be removed from our SNP pool. In total, 2,324,569 BMI-analysed SNPs remained in the study (see Table 1).

**Table 1:** SNP classification for the analyses of the BMI GWAS data.

Analysis	Group classification	Number of SNPs
P-value based analysis	Significant (P < 5 × 10^−8^)	40,835
	Non-significant (P ≥ 5 × 10^−8^)	2,283,734
Sensitivity analysis	Significant (P < 5 × 10^−8^)	40,835
	Non-significant (P ≥ 5 × 10^−7^; excluded SNPs with 5 × 10^−8^ < P < 5 × 10^−7^)	2,269,795
	Non-significant (P ≥ 5 × 10^−6^; excluded SNPs with 5 × 10^−8^ < P < 5 × 10^−6^)	2,248,179
	Non-significant (P ≥ 5 × 10^−5^; excluded SNPs with 5 × 10^−8^ < P < 5 × 10^−5^)	2,215,048
Linkage disequilibrium (LD) analysis	Significant (within a 1 Mb region)	805,908
	Non-significant (not within a 1 Mb region)	1,518,661
	Significant (in regions of high LD)	77,104
	Non-significant (not within regions of high LD)	2,247,465

Following the processing of data by removing SNPs with ambiguous information and mappings, the analyses of the BMI GWAS data^9^ were split into three sub-analyses. The initial analysis and its SNP set assignment was based on the general GWAS P-value threshold of 5 × 10^−8^. The ensued sensitivity analysis aimed for a more stringent group delineation between significant and non-significant SNPs by excluding SNPs whose P-value fell within a certain range. Each non-significant group was separately analysed in relation to the initial significant SNP group (P < 5 × 10^−8^). Considering two distinct approximations for the LD of SNPs, non-significant variants were added to the respective significant group if they were either located within a 1 Mb region adjacent to a significant SNP or if they are located in known regions of high LD (as extracted from plinkQC^70^).

**Figure 1:**
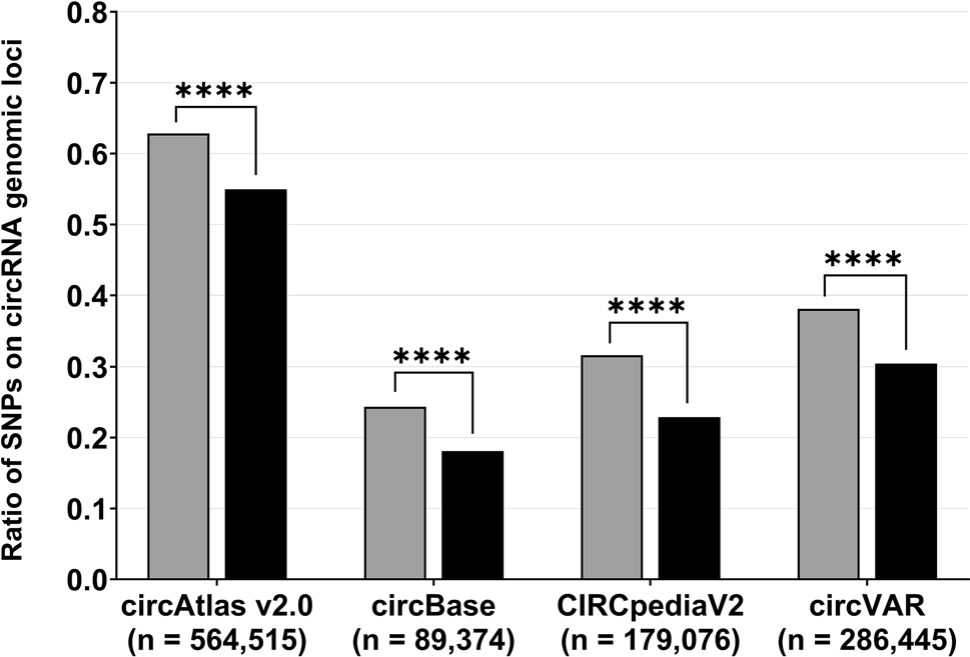
**Genome-wide significant SNPs for BMI are enriched in genomic loci of circRNAs.** SNPs were classified as significant and non-significant based on the genome-wide P-value threshold of 5 × 10^−8^. Correspondingly, SNPs with a lower P-value than this threshold were considered significant (grey; total of 40,835 SNPs), while SNPs with P-values exceeding this cut-off were defined as non-significant (black; total of 2,283,734 SNPs). Genomic positions of these SNPs were checked if they matched the genomic coordinates of circRNAs extracted from the databases circAtlas v2.0 (GRCh38), circBase, CIRCpediaV2 and circVAR using a custom R script. The numbers shown in the parentheses indicate the number of circRNAs included in the respective dataset (see also Supplementary Table S1). The results of the statistical tests can be found in Supplementary Table S5. ****P < 0.0001; *SNP* single nucleotide polymorphism.

We observed a significant and consistent enrichment of genome-wide significant SNPs (P < 5 × 10^−8^, see Table 1) for BMI on circRNA genomic loci throughout all circRNA datasets (see Fig. 1 and Supplementary Table S5). Thus, 62.86% (25,669 SNPs) of all significant SNPs were present on at least one circRNA genomic locus included in circAtlas v2.0 (GRCh38), while the proportion of non-significant SNPs on circRNAs was significantly lower (54.96%; P < 0.0001; OR = 1.39; 95% CI = [1.36, 1.42]). Analogous findings were detected in the datasets of circBase, CIRCpediaV2 and circVAR. Genomic loci of circRNAs included in circBase harboured 24.36% of significant SNPs (9,949 SNPs; P < 0.0001; OR = 1.46; 95% CI = [1.43, 1.49]), while circRNAs extracted from CIRCpediaV2 and circVAR encompassed 31.62% (12,913 SNPs; P < 0.0001; OR = 1.56; 95% CI = [1.53, 1.59]) and 38.11% (15,561 SNPs; P < 0.0001; OR = 1.41; 95% CI = [1.38, 1.44]) of significant BMI-SNPs, respectively. The ratio of non-significant SNPs within the genomic coordinates of circRNAs remained significantly lower (see Fig. 1 and Supplementary Table S5).

### Sensitivity analysis reveals marginal deviations

**Figure 2:**
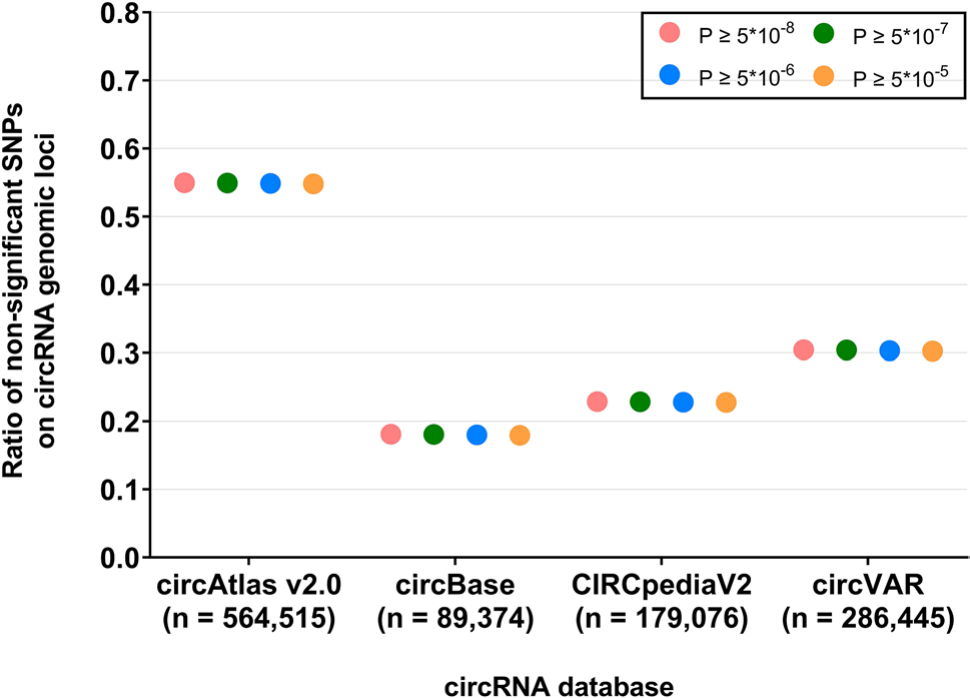
**Sensitivity analysis reveals deviations of ratios of circRNA-located non-significant SNPs.** To obtain a more distinct group delineation between significant and non-significant BMI-SNPs within the sensitivity analysis, the non-significant SNPs (P ≥ 5 × 10^−8^; pink) were re-classified applying the additional P-value cut-offs of 5 × 10^−7^ (green), 5 × 10^−6^ (blue) and 5 × 10^−5^ (orange). Non-significant SNPs with P-values ranging between these thresholds and the initial threshold of 5 × 10^−8^ were removed from the respective group. The results were obtained by applying the custom R script to the newly defined dataset of non-significant SNPs and were compared to the initial group of significant SNPs (P < 5 × 10^−8^). The respective statistical outcomes are stated in Supplementary Table S6. *SNP* single nucleotide polymorphism.

Next, we tested the sensitivity of our analysis by classifying the non-significant SNPs in accordance with three newly defined thresholds (P ﻿≥ 5 × 10^−7^, P ﻿≥﻿ 5 × 10^−6^ and P ﻿≥﻿ 5 × 10^−5^). Despite a stricter definitional delineation, we observed solely marginal deviations in ratios of non-significant SNPs located on circRNA genomic loci (see Fig. 2 and Supplementary Table S6). Accordingly, we still detected a statistically significant enrichment of genome-wide significant BMI variants on the genomic loci of circRNAs of all databases when re-classifying the group of non-significant SNPs (see Supplementary Table S6).

### Defining approximations of the linkage disequilibrium of SNPs does not affect the outcome

**Figure 3:**
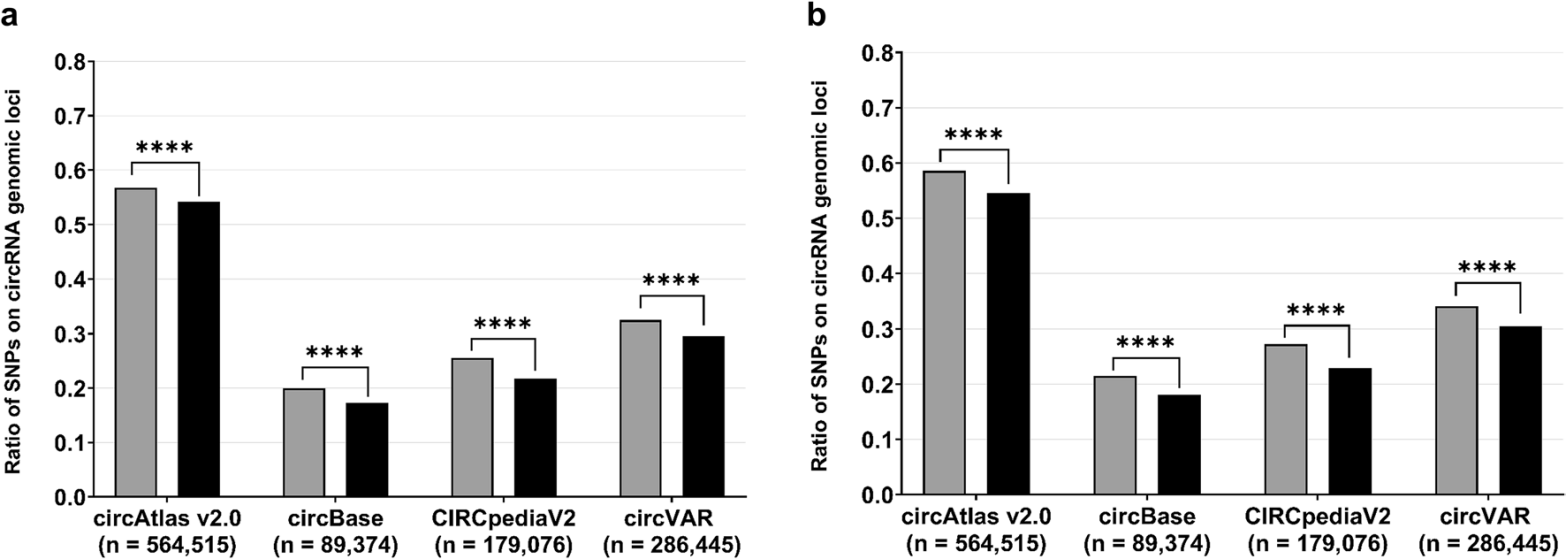
**Consideration of the linkage disequilibrium of BMI-SNPs located on circRNA genomic loci.** (**a**) To correct for the LD of SNPs, we have defined non-significant SNPs (black; total of 1,518,661 SNPs) that were located within a 1 Mb region adjacent to a significant SNP (P < 5 × 10^−8^) as a ‘significant region’ (grey; total of 805,908 SNPs) as well. (**b**) Further, in an additional LD approximation, non-significant variants (black; total of 2,247,465 SNPs) located within an already described region of high LD (as stated in plinkQC^70^) were classified as ‘significant’ (grey; total of 77,104 SNPs). The corresponding statistical results can be obtained from Supplementary Table S7. ****P < 0.0001; *SNP* single nucleotide polymorphism.﻿

Non-significant SNPs can be located within linkage disequilibrium (LD) regions comprising significant variants^26,27^. By defining approximations for the LD structures of the BMI-associated SNPs, we obtained an overall increase in the number of ‘significant’ variants and a decrease in the quantity of non-significant SNPs (see Table 1). Remarkably, by applying these newly defined groups to our R script, we detected similar results as in our previous examinations. Again, the genome-wide significant SNPs were more frequently located on circRNA genomic loci than non-significant variants, regardless of whether the LD was approximated by a 1 Mb region adjacent to significant SNPs or by known regions of high LD (see Fig. 3 and Supplementary Table S7).

### Enrichment of genome-wide significant SNPs is not a general characteristic

Given the observed enrichment of genome-wide significant BMI-SNPs on genomic loci of circRNAs, we aimed to uncover whether this localisation is a BMI-specific feature or if it is a general phenomenon. Therefore, we extracted data of additional GWAS studies analysing arbitrary, but distinct phenotypes (see Supplementary Tables S2 and S3). As circRNAs are generally highly expressed in the brain and especially during neurogenesis^16^, we assumed that this SNP enrichment might be an effect exclusively seen for traits associated with the central nervous system (CNS). To investigate a widespread range of traits, we have chosen GWAS datasets pertaining to one anthropometric trait (body height^9^), two neurological conditions (amyotrophic lateral sclerosis^28^ and epilepsy^29^), four peripheral diseases (chronic kidney disease^30^, heart failure^31^, pernicious anemia^32^ and ulcerative colitis^33^) and two psychiatric disorders (anorexia nervosa^34^ and autism spectrum disorder^35^; see Supplementary Tables S2 and S3). As we identified consistent results throughout the four circRNA datasets for BMI and since GWAS data is predominantly provided in the GRCh37 genome version, we decided to exclusively analyse the additional SNP datasets with the circRNAs extracted from circBase. Therefore, all prior performed processing steps are omitted.

**Figure 4:**
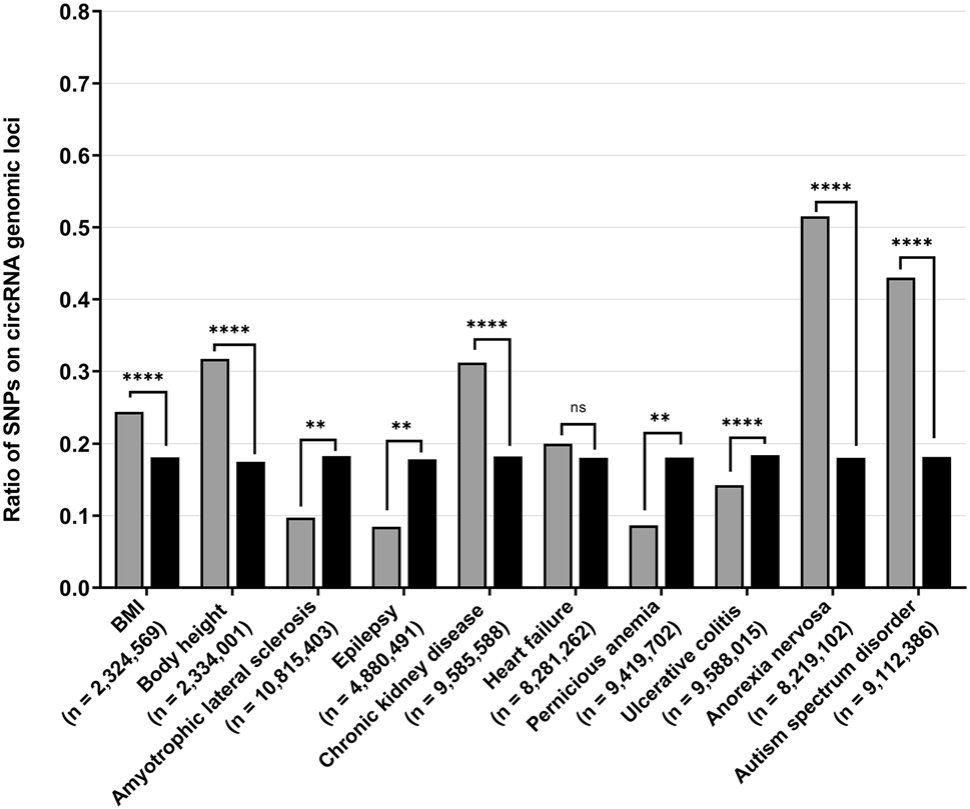
**Enrichment of genome-wide significant SNPs is not a cross-phenotype feature**. To test whether the detected enrichment of genome-wide significant SNPs (P < 5 × 10^−8^) is BMI-specific or a general characteristic, we extracted datasets of additional GWAS studies analysing various phenotypes. We have selected one anthropometric trait (body height^9^), two neurological diseases (amyotrophic lateral sclerosis^28^ and epilepsy^29^), four peripheral diseases (chronic kidney disease^30^, heart failure^31^, pernicious anemia^32^ and ulcerative colitis^33^) as well as two psychiatric disorders (anorexia nervosa^34^ and autism spectrum disorder^35^). The results of the BMI GWAS are presented as a reference and are exclusively based on the analysis with the circBase circRNA dataset. The corresponding statistical results are stated in Supplementary Table S8. ****P < 0.0001; **P < 0.01; *ns* not significant, *SNP* single nucleotide polymorphism.

A statistically significant enrichment of genome-wide significant SNPs (P < 5 × 10^−8^) was determined for SNPs from GWAS pertaining to both psychiatric disorders, namely anorexia nervosa (AN; P < 0.0001; OR = 4.83; 95% CI = [3.88, 6.00]) and autism spectrum disorder (P < 0.0001; OR = 3.40; 95% CI = [2.26, 5.12]), as well as the anthropometric trait of the body height (P < 0.0001; OR = 2.19; 95% CI = [2.17, 2.22]) and the peripheral chronic kidney disease (P < 0.0001; OR = 2.03; 95% CI = [1.82, 2.27]; see Fig. 4 and Supplementary Table S8). No significant difference of circRNA genomic loci localisation was detected for SNPs extracted from a heart failure GWAS (P = 0.38; OR = 1.14; 95% CI = [0.85, 1.51]; see Fig. 4 and Supplementary Table S8). The inverse circRNA enrichment of non-significant SNPs was detected for SNPs from GWAS studies analysing the neurological traits of amyotrophic lateral sclerosis (ALS; P = 0.003; OR = 0.48; 95% CI = [0.30, 0.78]) and epilepsy (P = 0.008; OR = 0.43; 95% CI = [0.22, 0.82]) as well as the peripheral diseases pernicious anemia (P = 0.003; OR = 0.43; 95% CI = [0.24; 0.76]) and ulcerative colitis (P < 0.0001; OR = 0.74; 95% CI = [0.69, 0.78]; see Fig. 4 and Supplementary Table S8).

Considering that a low BMI is also a characteristic of AN^36^, we additionally wanted to assess if significant SNPs localised on circRNAs overlap between AN and BMI. Of the 9949 significant BMI-SNPs located on circBase-extracted circRNAs (see Supplementary Table S5) and the 168 significant and circRNA-located AN-SNPs (see Supplementary Table S8), none overlapped (not shown).

### Deviations of significant BMI-SNPs on circRNAs genomic loci between females and males

**Figure 5:**
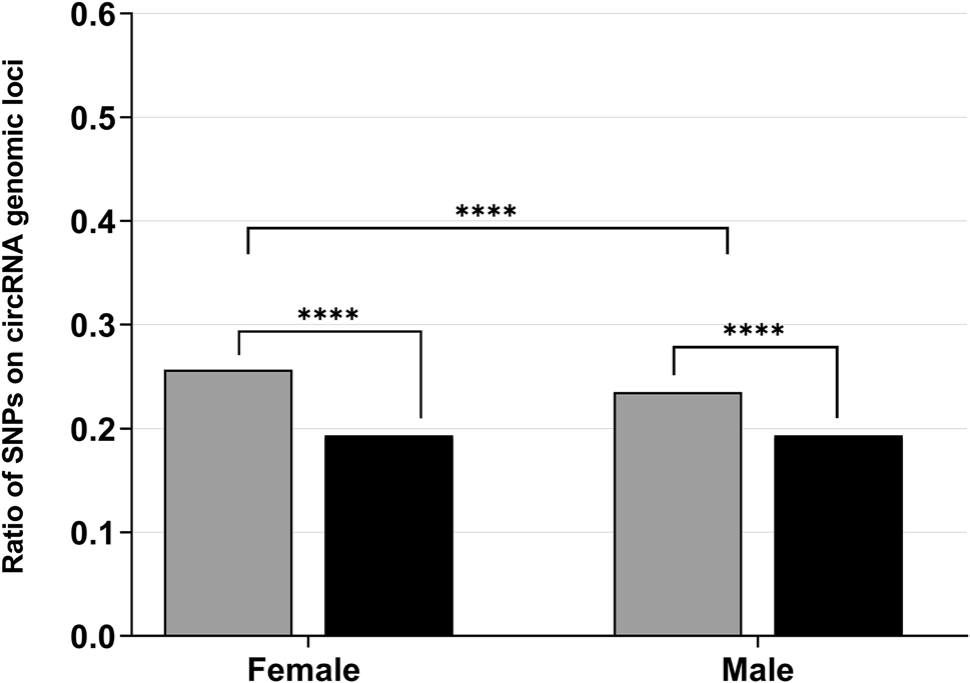
**Deviations of genome-wide significant BMI-SNPs located on circRNAs between females and males.** To explore whether the enrichment of significant BMI-associated SNPs deviates between females and males, we extracted data of a BMI-GWAS^37^ analysing both sexes separately. We applied the custom R script to the significant (grey; total of 27,653 SNPs in females; total of 22,833 SNPs in males) and non-significant (black; total of 27,352,598 SNPs in females; total of 27,357,420 SNPs in males) SNP data concomitantly with the circRNA data extracted from circBase separately for both sexes. Subsequently, we compared the number of circRNA-located significant SNPs for females against the quantity of SNPs encompassed in circRNA loci for males (Chi-square test). The statistical results are stated in Supplementary Table S9. ****P < 0.0001; *SNP* single nucleotide polymorphism.

To assess if the enrichment of significant BMI-SNPs on circRNA genomic loci applies equally to both sexes, we extracted data from an additional GWAS for BMI^37^ examining both sexes separately. More than 25% of the significant BMI SNPs for females are localised in at least one circRNA genomic locus included in circBase (7109 SNPs; see Fig. 5 and Supplementary Table S9). The proportion of significant SNPs for males was lower with 23.53% (5372 SNPs). For both sexes, the fraction of non-significant SNPs was identical at 19.35% (females: 5,292,550 SNPs; males: 5,294,287 SNPs). We were thus able to detect a significant enrichment of genome-wide significant variants for both, females (P < 0.0001; OR = 1.44; 95% CI = [1.40, 1.48]) and males (P < 0.0001; OR = 1.28; 95% CI = [1.24, 1.32]; see Fig. 5 and Supplementary Table S9). By comparing both sexes with each other, we detected a significant sex difference for the circRNA localisation of the genome-wide significant SNPs. BMI-associated SNPs significant in females are more abundant on circRNA genomic loci than the significant SNPs for BMI in males (P < 0.0001; OR = 1.13; 95% CI = [1.08, 1.17]; see Fig. 5 and Supplementary Table S10). Additionally, we were able to confirm this enrichment of BMI-associated SNPs in females in the largest dataset of circAtlas v2.0 (GRCh37; see Supplementary Fig. S1 and Supplementary Table S10).

Subsequently, we assessed whether circRNA-located SNPs significant in females and males overlap. We have found 2780 SNPs that were significant for both sexes and were localised on at least one circRNA (not shown). To obtain information about overrepresented functions and cellular compartments via a gene ontology (GO) analysis, we first mapped those overlapping SNPs to their corresponding genes using the Variant Effect Predictor (VEP) by Ensembl. The SNPs mapped to 198 unambiguous Ensembl gene IDs. The GO analyses revealed no significant overrepresentation for any biological process. By analysing the GO molecular functions terms, 160 of those genes were shown to be involved in protein binding (GO:0005515, FDR P = 4.83 × 10^−8^). Further, the 198 genes seem to be enriched in the nucleus (GO:0005634, FDR P = 5.89 × 10^−4^), membrane-bounded organelles (GO:0043227, FDR P = 6.36 × 10^−4^; GO:0043231, FDR P = 6.82 × 10^−4^), nuclear lumen (GO:0031981, FDR P = 8.25 × 10^−4^), intracellular organelles (GO:0043229, FDR P = 8.29 × 10^−4^), cytosol (GO:0005829, FDR P = 8.51 × 10^−4^), nucleoplasm (GO:0005654, FDR P = 1.27 × 10^−3^), intracellular anatomical structure (GO:0005622, FDR P = 1.54 × 10^−3^), organelles (GO: 0043226, FDR P = 2.20 × 10^−3^), cytoplasm (GO:0005737, FDR P = 6.30 × 10^−3^), membrane-enclosed lumen (GO:0031974, FDR P = 1.23 × 10^−2^), organelle lumen (GO:0070013, FDR P = 1.34 × 10^−2^; GO:0043233, FDR P = 1.46 × 10^−2^) and cytoplasmic stress granules (GO:0010494, FDR P = 2.59 × 10^−2^).

### Interactions of microRNAs (miRNAs) and circRNAs

As it is known that circRNAs often function as miRNA sponges^14,38^, we aimed to explore potential interactions between circRNAs harbouring significant BMI-SNPs and miRNAs. Thus, we downloaded data from the Encyclopedia of RNA Interactions (ENCORI)^39^ containing 475,341 miRNA target sites (GRCh37) of 643 miRNAs that were correlated with various circBase circRNAs. In total, 5717 circRNAs from the circBase database contained at least one of the 9949 significant SNPs for BMI (see Supplementary Table S5). Of these, 4100 (71.72%) were linked to at least one miRNA target site (not shown). The 20 most miRNA-comprising circRNAs by raw numbers were on average 1,243,002 bp long. To be able to estimate more precisely the number of miRNA binding sites presumably present in a given circRNA, we have normalised the number of miRNA targets found for a specific circRNAs on its length (see Supplementary Fig. S2b). Thereby, hsa_circ_0005103 showed the highest ratio of miRNAs based on its length (0.1449; see Supplementary Fig. S2b), while hsa_circ_0007319 showed the highest raw count of miRNA target sites (4275; see Supplementary Fig. S2a). 641 miRNAs (99.69%) could be linked to a circRNA containing at least one BMI-SNP. Of these, two of the miRNAs with the highest numbers of circRNAs linked (hsa_miR-15a-5p and hsa_miR-16-5p; see Supplementary Fig. S3) have been implicated in obesity-related traits^40–43^.

### Experimental validation of the SNP and a circRNA candidate

Next, to assess whether BMI-associated SNPs might affect the level of circRNAs, we selected one genome-wide significant SNP for BMI located on a circRNA. Accordingly, we screened for a genome-wide significant SNP detected in the BMI GWAS^9,44^ that is prospectively localised on a circRNA extracted from circBase, while further fulfilling predefined requirements. 9949 BMI-associated SNPs were found to be located on circRNAs included in circBase (see Supplementary Table S5). Of these, 5471 variants exhibited a MAF of at least 30% and 1749 SNPs were further detected in regions of circRNAs previously confirmed in multiple cell lines and tissues. After the validation of the set distance between SNP and circRNAs’ backsplice junction (BSJ) and LD structures, the SNP rs4752856 (chr11: 47,648,402; GRCh37; MAF = 0.3526; P = 9.4 × 10^–46^)^9^ whose risk allele (A) predisposes to an increased BMI (β = 0.0242)^9^ was selected. It is located on the mitochondrial carrier homologue 2 gene (*MTCH2*)-derived circRNA hsa_circ_0022025 (chr11: 47,647,226–47,648,679; GRCh37). By genotyping 21 recruited participants (age: 24.95 ± 3.46 years old; BMI: 20.77 ± 1.77 kg/m^2^), we identified that ten probands were heterozygous (G/A), ten were homozygous for G/G and one was homozygous for A/A. The Hardy–Weinberg-Equilibrium was thus fulfilled.

### Allelic levels of rs4752856-containing circRNAs are skewed in favor of the risk allele

**Figure 6:**
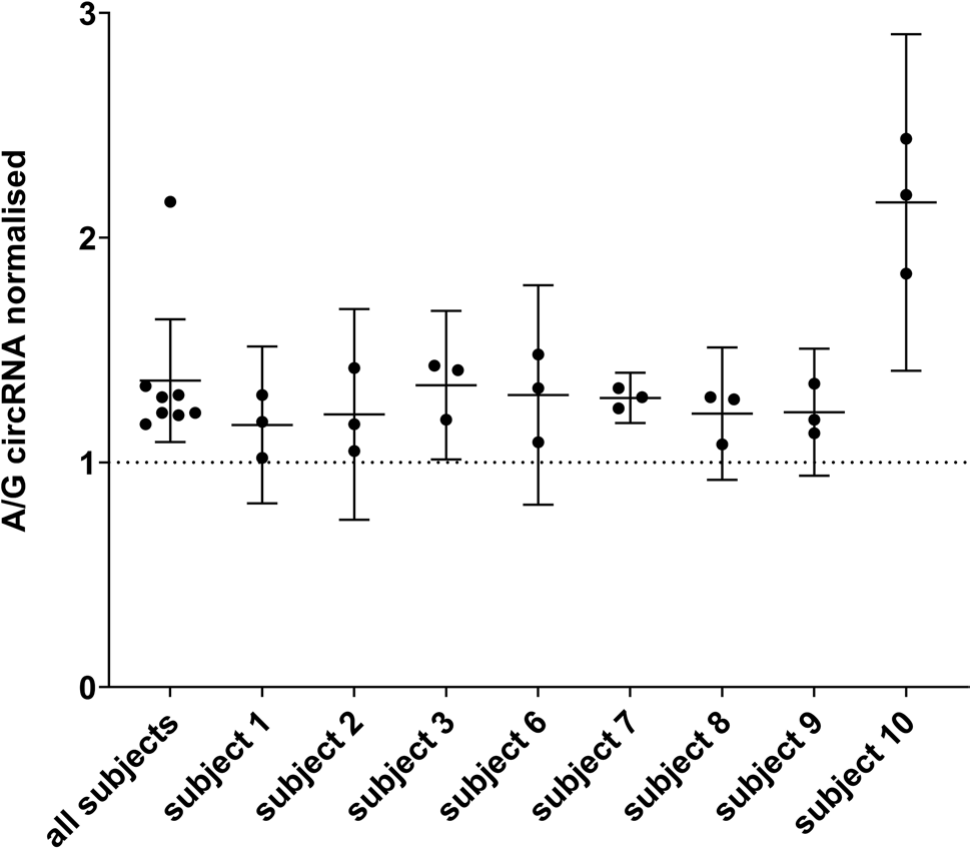
**Allelic ratios of hsa_circ_0022025 in eight heterozygous individuals for SNP rs4752856.** Primer extension analysis of the allelic levels was performed with the ABI Prism SNaPshot Multiplex kit (Applied Biosystems, Foster City, CA, USA) as previously described^45^. The assay was performed in triplicates for each subject indicated by the single data points. For ‘all subjects’, each data point refers to the mean value of one subject. The allelic circRNA ratios were normalised with the allelic gDNA ratios. Besides the mean value (middle bar), the upper and lower limits of the 95% confidence interval are shown. The descriptive statistics of the normalised results were analysed with SPSS (version 28.0.0.0) and are stated in Supplementary Table S11. *circRNA* circular RNA.

To determine the relative levels of the circRNAs derived from the G- and A-allele of rs4752856, we performed highly sensitive primer-extension assays (SNaPshot)^45^ on the genomic DNA (gDNA) and circRNA-derived complementary DNA (cDNA) of heterozygous individuals. The gDNA was used to normalize the circRNA values. For each sample, the assay was performed in triplicates. Homozygous samples acted as assay controls and were not further analysed (see Supplementary Figs. S4 and S5). Two samples of heterozygous individuals were excluded from subsequent statistical analysis due to low signals and high background noise. Thus, eight heterozygous samples remained in the study (for exemplary output see Supplementary Fig. S6). All of these samples revealed a distinct skew in favor of the BMI-increasing allele (A-allele; see Fig. 6 and Table 2). The one-sample Wilcoxon test showed that the normalised circRNA value was significantly higher (observed median = 1.25; asymptotic two-sided P-value = 0.012; effect size r = 0.63; see Supplementary Table S11) than the hypothesized median value of 1. Collectively, we ascertained that the A-allele of rs4752856 was 25% more abundant on the circRNA than the G-allele (see Fig. 6 and Table 2).

**Table 2:** Allelic ratios of the SNP rs4752856 on the circRNA hsa_circ_0022025 and the genomic fragment of *MTCH2*.

Subject	Triplicate I			Triplicate II			Triplicate III		
	A/G circRNA	A/G gDNA	Normalised circRNA/gDNA	A/G circRNA	A/G gDNA	Normalised circRNA/gDNA	A/G circRNA	A/G gDNA	Normalised circRNA/gDNA
Subject 1	0.61	0.59	1.02	0.65	0.55	1.18	0.76	0.58	1.30
Subject 2	0.62	0.59	1.05	0.66	0.56	1.17	0.80	0.56	1.42
Subject 3	0.70	0.59	1.19	0.78	0.55	1.41	0.80	0.56	1.43
Subject 6	0.65	0.60	1.09	0.84	0.57	1.48	0.75	0.56	1.33
Subject 7	0.74	0.57	1.29	0.71	0.57	1.24	0.79	0.59	1.33
Subject 8	0.62	0.57	1.08	0.70	0.54	1.29	0.75	0.58	1.28
Subject 9	0.69	0.62	1.13	0.80	0.60	1.35	0.72	0.60	1.19
Subject 10	1.08	0.59	1.84	1.37	0.56	2.44	1.25	0.57	2.19

The A/G ratios of the primer extension products were determined for both the circRNA-derived cDNA as well as genomic DNA (for details see “Materials and methods” section). The circRNA ratios were normalized with regard to the genomic DNA. *A* risk allele, *G* major allele, *circRNA* circular RNA, *gDNA* genomic DNA.

## Discussion

Our study aimed to analyse the putative interactions of SNPs affecting BMI variation and circRNAs. Therefore, we initially investigated whether genome-wide significant SNPs (P < 5 × 10^−8^) for BMI are more frequently located on genomic loci harbouring circRNAs than non-significant SNPs (P ≥ 5 × 10^−8^). We indeed identified a significant enrichment of genome-wide significant BMI-associated SNPs on circRNA genomic loci extracted from the databases circAtlas v2.0, circBase, CIRCpediaV2 and circVAR. This enrichment was consistent throughout the four circRNA datasets. Concurrently, our sensitivity analysis revealed analogous findings. Despite the exclusion of SNPs with P-values within a certain range and a resultant stricter delineation between the definitions of significant and non-significant SNPs, we still detected the previously shown enrichment of significant SNPs. Equally, this clustering was still evident when considering the LD structures of SNPs, regardless of whether a 1 Mb region was defined as an LD block or whether already known regions of high LD were analysed.

A previous study observed that SNPs with the highest impact on circRNAs, so-called circRNA quantitative trait loci (circQTLs), were mainly located in the flanking sites of the respective circRNAs, while merely a limited proportion reside inside the circRNAs. By analysing various sequence-defined elements, an intron-based enrichment was detected^24^. As GWAS signals predominantly map to non-coding regions^6,7,10,11^ and as we exclusively analysed the genomic loci of circRNAs based on the start and stop positions deposited in the respective databases, and thereby neglecting splice isoforms of circRNAs, our results might be driven by this intron-specific enrichment of the significant SNPs. Consequently, we may have overestimated the number of SNPs that are potentially located on the matured circRNAs. However, this equally applies to significant and non-significant variants. Yet, SNPs in the flanking introns exerting their effects on circRNAs as proposed by Liu et al.^24^ might at least be partially covered by our genomic level-based analysis. Still, since we cannot utilize the circRNA database data to infer which splice forms are generally formed, our approach can potentially account for the majority of isoforms putatively generated. Conversely to the intronic circQTL enrichment, an additional report showed that circQTLs were less likely to be intronic variants and instead tended to be localised within 5' untranslated regions and exonic regions^46^, concurring with the fact that the majority of circRNAs are comprised of exons^24^. Nevertheless, both studies have demonstrated that circQTLs with a high influence on circRNAs are more frequently located on disease-associated loci than SNPs that do not influence the circRNA^24,46^. Although we assigned the SNPs significance level based on the P-value and we thus might have encompassed non-causal variants in the significant group, it is certainly feasible that the majority of our significant SNPs localized to circRNAs may have a detectable influence on these circular transcripts. Generally, genetic variants located within genomic loci of circRNAs could putatively alter sequences of binding sites for miRNAs and RNA-binding proteins (RBPs), albeit Thomas and Sæstrom^47^ ascertained that the SNP density at these binding sites is generally diminished in circRNAs. Yet, as circRNAs exhibit a cell type specific expression^17^, it is still plausible that while there is generally a reduced enrichment of SNPs in these regions, this may vary depending on the cell type from which the circRNA was extracted. Still, an altered binding affinity of circRNAs towards their targets can have profound consequences, which remain to be validated in subsequent in vitro and in vivo studies.

As we observed a significant accumulation of the genome-wide significant SNPs for BMI on the genomic loci of circRNAs, we subsequently aimed to explore whether this enrichment was evident for SNPs associated with additional diseases and traits. As a previous report has demonstrated that circRNAs are highly enriched in the brain and synapses^16^, we postulated that the detected enrichment might be associated with CNS-mediated diseases and traits. Replication of our analyses with GWAS data pertaining to anthropometric, neurological, peripheral, and psychiatric phenotypes revealed a significant clustering of genome-wide significant SNPs for the psychiatric disorders (AN and autism spectrum disorder), the anthropometric measure of the body height and the peripheral CKD. The inverse enrichment of non-significant variants was detected for SNPs extracted from GWAS pertaining ulcerative colitis, pernicious anemia and the two neurological diseases (ALS and epilepsy). No significant deviation in circRNA localisation of significant and non-significant variants was ascertained for variants analysed in a GWAS for heart failure. Consequently, we could not confirm our hypothesis. Apparently, this enrichment is not a general feature of circRNAs. Clearly, the results may be biased due to the characteristics and implementations of the GWAS. The GWAS for BMI and body height, for instance, were conducted by the same authors^9^ and given the definition of the BMI (kg/m^2^), we cannot exclude the possibility that the results obtained for the BMI GWAS were driven by the results of the dataset pertaining the body height. Generally, a high polygenicity for BMI and body height has been reported^48^. Further, genetic correlations between the phenotypes may have impacted our results. For example, past studies of our research group identified nine SNP alleles at three independent genetic loci that were associated with both, AN, and BMI. We were able to show that all nine AN susceptibility alleles concomitantly accounted for a lower BMI^49^. This correlation was confirmed by additional studies^50–52^. Yet, we did not assess any overlapping significant SNPs for BMI and AN located on circRNAs and consequently, could exclude SNP-based bias. Still, correlation and reciprocal interference between our results regarding BMI and AN cannot be fully excluded as our sex-specific analysis based on data from the BMI GWAS of Pulit et al.^37^ implies that genome-wide significant BMI-SNPs in females are more abundant on circRNA genomic loci than significant BMI-SNPs in males. Since it is known that AN patients are predominantly female^53^ and SNPs associated with a higher risk of AN predispose to a lower BMI^49–52^, we can assume that the BMI-SNPs on circRNAs mainly predispose to a lower BMI and that the effect on the etiology of obesity is marginal. In addition, most phenotypes analysed have already been associated with circRNAs^54–59^. For example, circRNA expression profiling of post-mortem brains of autistic patients and controls identified 60 circRNAs and more than 8000 autism-associated circRNA-miRNA-mRNA interactions. It was shown that targets of these axes were mainly risk genes for autism. Some of these high-risk genes were even modulated by upregulated circRNAs acting as a miR-204-3p sponge in human neuronal cells^54^. Merely studies on AN and pernicious anemia and their circRNA implication are lacking. Yet, it is unknown how disease-associated SNPs affect these mechanisms.

Previously, most circRNAs were functionally characterised as miRNA sponges^14,38^. Consequently, we have explored putative miRNA-circRNAs interactions based on a dataset extracted from the ENCORI database^39^. More than 70% of all circBase-extracted circRNAs were found to be linked to a least one miRNA. Yet, the circRNAs with the highest raw counts of miRNA target sites tended to be large in size. We assume that these circRNAs do not in fact span the entire range between predicted start and stop position stated in the database, but are indeed smaller. Therefore, we performed a normalisation based on the circRNA length. This yielded low ratios of miRNA target sites in contrast to the circRNAs length.

Almost all miRNAs included in the ENCORI dataset were determined to be correlated to SNP-harbouring circRNAs. Within the five miRNAs linked to the highest number of circRNAs, two were associated with an obesity-related trait. Interestingly, hsa-miR-15a-5p has been reported to be involved in the process of adipocyte differentiation^40^ and was differentially expressed in response to a low-fat diet^41^. Similarly, hsa-miR-16-5p was found to be downregulated after bariatric surgery^42^ and after aerobic exercise training^43^. It is thus feasible, that certain circRNAs linked to BMI-associated SNPs might act on miRNAs as well.

Notably, due to predictions of circRNAs being based on computational algorithms and a lack of experimental validation, the possibility arises that the circRNAs from the databases represent false positives^60^. In general, given the high number of SNPs included in this study, the statistical power increased substantially and thus might exaggerate the potentially clinically negligible effects^61^.

To analyse a potential functional effect of SNPs on circRNAs obtained by our in silico analyses, we selected a BMI-increasing SNP (rs4752856) located on a *MTCH2*-derived circRNA (hsa_circ_0022025) for follow-up in vitro analyses. Using a highly sensitive primer extension assay, we determined the relative levels of the circRNAs derived from the G- and A-allele of heterozygous probands. We detected a significant skewing in favour of the risk allele (A). Normally, when investigating *cis*-regulatory effects, a large sample pool is required. But as we exclusively analysed heterozygous individuals, the respective other allele acted as the internal control^45^ warranting our small number of eight probands.

Previous studies have already demonstrated that SNPs have a considerable impact on the circRNAs’ levels^22,23^. Zhou et al. showed that SNP rs12196996, which was significantly correlated with an increased risk to develop coronary artery disease (CAD), was associated with decreased circFOXO3 levels in individuals with the GG genotype. It has been suggested that the increased CAD risk is caused by the effect of the SNP on circFOXO3^23^. Further, it is known that the levels of circRNAs in adipose tissue are different between obese and lean individuals^18,19^. One circRNA, circSAMD4A, exhibited a positive correlation with BMI in obese patients which was assumed to exert its effects through interactions with miR-138-5p, ultimately regulating the expression of *EZH2*^19^. Further, a deep sequencing analysis discovered thousands of circRNAs within the adipose tissue, which were dynamically regulated during adipogenesis and in obesity. A downregulation of circRNAs in contrast to the linear mRNAs was detected in obese mice^18^.

*MTCH2* is known to be an obesity susceptibility gene^62,63^. Previously, it has been positively correlated with BMI variance and was reported to be upregulated during adipogenesis^62,64^. Elevated *MTCH2* expression levels have been determined in obese women^62^. Given that circRNAs can regulate mRNA levels due to their role as miRNA sponges^14,38^ and given that we have detected increased levels of the hsa_circ_0022025 derived from the BMI-increasing allele (A) of rs4752856, it is feasible that this circRNA may act as a miRNA sponge indirectly affecting the *MTCH2* mRNA levels. Additionally, MTCH2 protein levels are increased in obese individuals^62^, which might potentially reflect the increased mRNA levels. Still, it has been reported that circRNAs can undergo cap-independent translation^14^. Thus, our detected increase of hsa_circ_0022025 derived from the risk allele (A) might hint at such a cap-independent translation, consequently raising the protein levels of MTCH2. Further, animal models have shown that a knockout of *Mtch2* leads to a lower susceptibility for weight gain^63^. These transgenic mice exhibited elevated levels of energy expenditure^63^, while a loss of Mtch2 was shown to be protective for diet-induced obesity^65^. Hence, rs4752856’s BMI-increasing effect^9^ might be caused by the here detected increased levels of hsa_circ_0022025.

Accordingly, it is indeed conceivable that BMI-affecting SNPs, such as the here examined rs4752856, might exert an impact on circRNAs and ultimately affect the BMI of the risk allele carriers. Yet, as circRNAs are cell-type specifically expressed^17^, a replication of the analysis using circRNAs from different sources could yield in different outcomes and since we solely examined one SNP on one circRNA, the obtained results merely serve as an indication for putative SNP-mediated mechanisms and should be confirmed in additional analyses of multiple circRNA-located and BMI-affecting variants.

Taken together, we have determined a significant enrichment of genome-wide significant SNPs for BMI in circRNAs in comparison to non-significant variants. A sensitivity analysis as well as approximations of LD structures revealed similar outcomes. Yet, we did not ascertain evidence that this enrichment is consistent throughout various phenotypes. Our analysis of the BMI-increasing SNP rs4752856 located on the *MTCH2*-derived circRNA hsa_circ_0022025 revealed a higher abundance of the risk (A) allele on the circular transcript compared to the major (G) allele. Thus, our analyses extend the current knowledge of interactions of SNPs with circRNAs providing evidence for effect of BMI-affecting SNPs on circRNAs and thus implications in obesity. Further analyses need to determine the extent of the here detected findings.

## Materials and methods

### Datasets of circRNAs and GWAS SNPs

The circRNA datasets were downloaded from four publicly available circRNA databases, namely circAtlas v2.0^66^, circBase^67^, CIRCpediaV2^68^ and circVAR^69^ (see Supplementary Table S1). All data was extracted from the download section of the respective database and contained exclusively human data. Data with ambiguous or incomplete annotations and circRNAs derived from genomic loci of the sex chromosomes were excised. The data was further checked for internal database duplicates.

The SNP summary statistics of the meta-analysis of BMI GWAS conducted by Yengo et al.^9^ was downloaded from the Genetic Investigation of Anthropometric Traits (GIANT) consortiums’ website (see Supplementary Table S2). Given the inconsistent assignment of reference genomes amongst the circRNA databases (see Supplementary Table S1), the SNP coordinates were shifted from GRCh37 to GRCh38 using the remapping service of the National Center for Biotechnology Information (NCBI) (https://www.ncbi.nlm.nih.hov/genome/tools/remap). As the lift over of certain SNPs was inconclusive, the respective variants were excluded. In total, 2,324,569 SNPs remained in the analysis (see Supplementary Tables S2 and Table 1).

In order to replicate the analysis for further phenotypes, additional GWAS data for ALS^28^, AN^34^, autism spectrum disorder^35^, body height^9^, CKD^30^, epilepsy^29^, heart failure^31^, pernicious anemia^32^ and ulcerative colitis^33^ were downloaded (see Supplementary Table S2). Putative gender deviations were analysed with data of an additional BMI GWAS^37^. All GWAS data was acquired in the GRCh37 genome version and was exclusively analysed pertaining the autosomal chromosomes.

### SNP classification

To assess whether genome-wide significant SNPs for BMI show a higher abundance on the genomic loci of circRNAs than non-significant variants, SNPs were assigned as significant (P < 5 × 10^−8^) or non-significant (P ≥ 5 × 10^−8^) based on their P-value (see Table 1).

Next, a sensitivity analysis was implemented intended to provide a more stringent delineation between the groups of SNPs from the BMI GWAS. Three additional P-value thresholds were introduced (5 × 10^−7^, 5 × 10^−6^, 5 × 10^−5^). The SNPs with P-values between 5 × 10^−8^ and the corresponding novel cut-off were excluded (see Table 1). Accordingly, while the group of significant SNPs remained unchanged, we obtained three diminished sets of non-significant variants (P ≥ 5 × 10^−7^, P ≥ 5 × 10^−6^ or P ≥ 5 × 10^−5^).

Subsequently, approximations of the LD structures of the BMI-SNPs were defined. Hence, all non-significant SNPs within a 1 Mb region adjacent to significant SNPs were assigned as significant as well (see Table 1). Since this definition of a 1 Mb region as a LD block was expected to yield a large number of false positives, the analysis of known regions of high LD as represented in plinkQC^70^ (https://github.com/meyer-lab-cshl/plinkQC/blob/master/inst/extdata/high-LD-regions-hg38-GRCh38.txt) was ensued. If a region of high LD contained one of the significant SNPs (based on the P-value), all non-significant SNPs within this region were also classified as significant (see Table 1).

The group assignment of SNPs derived from GWAS of additional phenotypes is shown in Supplementary Table S3.

### Enrichment analysis

A custom R script (R version: 4.0.5; RStudio version 1.4.1106) was applied to match the genomic coordinates of the circRNAs with those of the GWAS SNPs. The genomic coordinates of the circRNAs were constructed based on the predicted start and stop positions provided in the respective circRNA database. The total number of unique SNPs located on the genomic loci of circRNAs was extracted from the generated output. Subsequently, a two-sided Chi-square test and the odds ratio (OR) were calculated using GraphPad Prism (version: 9.2.0). The confidence intervals (CI) of the OR were computed using the Woolf logit intervals. The confidence level was set to 95%.

### Gene mapping and gene ontology analyses

To perform a functional assignment of SNPs, the rsIDs of these were initially allocated to the corresponding Ensembl gene IDs using the Ensembl Variant Effect Predictor (https://ensembl.org/Tools/VEP). Next, the gene IDs were analysed by the means of the Gene Ontology (http://www.geneontology.org) to yield functional classifications. Therefore, the PANTHER overrepresentation test was subjected applying the GO Ontology database. All 20,589 human genes listed in the database were used as a reference. As a statistical test, the Fisher’s exact test with a False Discovery Rate (FDR) correction was applied. Significance was given with FDR P < 0.05.

### In silico analyses of interactions between miRNAs and circRNAs

To analyse whether the circRNAs harbouring significant SNPs for BMI might interact with miRNAs, a dataset containing information regarding miRNA-circRNA interactions supported by Ago CLIP-sequencing data was downloaded from the ENCORI database (https://starbase.sysu.edu.cn)^39^. This contained 475,341 target sites of 643 miRNAs known to be linked to circRNAs. The data was based on the GRCh37 genome version. Using a marginally modified version of the custom R script implemented before, it was checked whether the circBase ID of circRNAs containing BMI-SNPs was included in the ENCORI dataset.

### Experimental validation of SNP effects

To determine the relevance of BMI-affecting SNPs on circRNAs, a genome-wide significant SNP for BMI (P < 5 × 10^−8^)^9,44^, whose circRNA localisation was predicted by the preceding in silico analysis, was selected for subsequent in vitro studies. Following conditions needed to be met: (1) the SNP is located on a circRNA that was verified to be expressed in multiple cell lines and tissues; and (2) the SNP exhibits a MAF of at least 30% to ensure the detection of heterozygous carriers required for the allelic expression assay (as described in^45^); and (3) the SNP is located in close proximity (< 2 kb) to the BSJ of the circRNA in order to amplify both BSJ and SNP on one fragment to exclude potential unspecific amplicons; and (4) is either a lead SNP or in high LD to a lead SNP.

### Study group

We collected blood of 21 healthy individuals (age: 24.95 ± 3.46 years old; BMI: 20.77 ± 1.77 kg/m^2^; 52.38% female). Written informed consent was given by each participant. This study was approved by the Ethics Committee of the Medical Faculty of the University Duisburg-Essen (15-6534-BO) and was performed in accordance with the Declaration of Helsinki.

### Genotyping

The DNA of each participant was isolated from whole-blood. Subjects were genotyped performing a PCR (Veriti 96-well thermal cycler; Applied Biosystems, Foster City, CA, USA) with SNP-specific gDNA primers (see Supplementary Table S4). Samples were purified with the QIAquick PCR Purification kit (Qiagen, Hilden, Germany) and sent for Sanger sequencing to Microsynth Seqlab GmbH in Göttingen, Germany. Sequence analysis and genotype assignment were performed by at least two experienced scientists using the SeqMan Pro software (version: v.10.1.0). Discrepancies were solved by either reaching consensus or by re-sequencing. The fulfilment of the Hardy–Weinberg-Equilibrium was checked.

### Experimental validation of the circRNA

Peripheral blood mononuclear cells (PBMCs) were isolated from the participants’ whole-blood applying a density gradient centrifugation utilizing Lymphoprep (Stemcell Technologies, Vancouver, BC, Canada) pre-filled Leucosep tubes (Greiner Bio-One GmbH, Frickenhausen, Germany) according to the manufacturer’s instructions. Total RNA was extracted from the isolated PBMCs using TRIzol (Invitrogen AG, Carlsbad, CA, USA). The manufacturer’s protocol was modified to increase the cell number and to minimize the dilution factor. Next, the total RNA samples were treated with at least 1 U RNase R (Epicentre Biotechnologies, Madison, WI, USA) per 1 µg RNA for 15 min at 37 °C. Subsequently, the samples were subjected to at least 2 U DNase I (New England Biolabs GmbH, Ipswich, MA, USA) per 10 µg RNA for 10 min at 37 °C followed by an EDTA (5 mM) heat inactivation step for 10 min at 75 °C. The remaining RNA was reversely transcribed to cDNA using the qScript cDNA SuperMix (Quanta Biosciences Inc., Beverly, MA, USA) as described in the manufacturer’s instructions. Residual linear contaminations were excluded for each sample by performing a PCR with primers spanning two exons including their in-between intron of the sex hormone binding globulin (*SHBG*; see Supplementary Table S4). Furthermore, the successful circRNA isolation was validated conducting a PCR with divergent primers amplifying the yippee-like 2 (*YPEL2*)-derived circRNA, hsa_circ_0005600. Divergent primers spanning the BSJ and the SNP (see Supplementary Table S4) were applied in a PCR (Veriti 96-well thermal cycler; Applied Biosystems, Foster City, CA, USA) on the circRNA-derived cDNA template to confirm the presence of both. A 2.5%-agarose gel electrophoresis validated the expected fragment size and was followed by purification of the PCR products (QIAquick PCR purification kit; Qiagen, Hilden, Germany) and Sanger sequencing (performed by Microsynth Seqlab GmbH, Göttingen, Germany). Sequence analysis and circRNA confirmation were conducted by at least two experienced scientists with the SeqMan Pro software (version: v.11.0.0). Discrepancies were solved by either reaching consensus or by re-sequencing. The desired fragments were successfully ascertained for all examined samples.

### Primer extension analysis

To determine the relative levels of hsa_circ_0022025, the amplification of the genomic DNA and circRNA-derived cDNA fragments for ten heterozygous individuals (G/A), two homozygous G/G probands, and one homozygous A/A participant was repeated in triplicates. The fragments were confirmed by a 2.5%-agarose gel and were subsequently purified (QIAquick PCR purification kit; Qiagen, Hilden, Germany). Next, the samples were subjected to a primer extension assay using the ABI Prism SNaPshot Mulitplex kit (Applied Biosystems, Foster City, CA, USA) and a specific SNaPshot primer (see Supplementary Table S4) following the manufacturer’s instructions. Equal amounts of cDNA and gDNA of each investigated participant were applied. Samples were heated for 3 min to 96 °C, followed by 25 cycles of 96 °C for 10 s, 53 °C for 5 s and 60 °C for 30 s. The SNaPshot reaction products were purified with shrimp alkaline phosphatase (GE Healthcare, Waukesha, WI, USA) and were analysed by gel capillary electrophoresis on the ABI Prism 3700 DNA Analyzer (Applied Biosystems, Foster City, CA, USA). The resultant electropherograms were examined using the GeneMapper 4.0 software by Applied Biosystem (Foster City, CA, USA). Next, allelic ratios of the circRNA-derived cDNA were normalised with the gDNA allelic ratios as in Eq. (1). The descriptive statistics with confidence intervals were based on the t-test and a one-sample Wilcoxon test and was conducted with SPSS (version: 28.0.0.0). The confidence level was set to 95%.

1 $$\frac{A{\text{-}}allele\, circRNA / G{\text{-}}allele\, circRNA}{A{\text{-}}allele\, gDNA/G{\text{-}}allele\, gDNA}$$

## Supplementary Information

Supplementary Information.

## Data Availability

The datasets generated and analysed during the current study are available in the Zenodo repository, 10.5281/zenodo.6726258. The sources of the used GWAS and circRNA datasets are stated in Supplementary Tables S1 and S2. Biological material can be obtained for research purposes. The R script can be obtained by the authors upon reasonable request.
