# Supplementary material for: Evidence for correlations between BMI-associated SNPs and circRNAs
Source: Sci Rep. 2022 Jul 25;12:12643. doi: 10.1038/s41598-022-16495-7 (PMC9314347; doi:10.1038/s41598-022-16495-7)
Supplement: Supplementary file 1 — Supplementary Information. [file 41598_2022_16495_MOESM1_ESM.pdf]

Evidence for Correlations Between BMI-Associated SNPs and circRNAs

Luisa Sophie Rajcsanyi[1,2]*, Inga Diebels[1], Lydia Pastoors[1], Deniz Kanber[3], Triinu Peters[1,2], Anna-Lena Volckmar[4], Yiran Zheng[1,2], Martin Grosse[3], Christoph Dieterich[5,6], Johannes Hebebrand[1,2], Frank J. Kaiser[3], Bernhard Horsthemke[3] and Anke Hinney[1,2]*

[1] Department of Child and Adolescent Psychiatry, Psychosomatics and Psychotherapy, University Hospital Essen, University of Duisburg-Essen, Essen, Germany.

[2] Center for Translational Neuro- and Behavioural Sciences, University Hospital Essen, Essen, Germany.

[3] Institute of Human Genetics, University Hospital Essen, Essen, Germany

[4] Institute of Pathology, University Hospital Heidelberg, Heidelberg, Germany

[5] Department of Internal Medicine III, University Hospital Heidelberg, Heidelberg, Germany

[6] German Center for Cardiovascular Research (DZHK), Partner site Heidelberg/Mannheim, Heidelberg, Germany

*****corresponding authors

Supplementary Materials

Supplementary Table S1: Overview of circRNA Datasets

Supplementary Table S2: Overview of Analysed GWAS Data

Supplementary Table S3: Assignment of SNPs of the GWAS Studies Analysing Additional Phenotypes

Supplementary Table S4: List of Used Primers

Supplementary Table S5: Results of the Analysis of BMI-SNPs Pertaining to their Localisation on circRNA Genomic Loci.

Supplementary Table S6: Results of the Sensitivity Analysis

Supplementary Table S7: Results When Considering the Linkage Disequilibrium of SNPs

Supplementary Table S8: CircRNA Location of SNPs Extracted from GWAS Pertaining Various Phenotypes

Supplementary Table S9: Gender-Separated Analysis of BMI-SNPs on the Genomic Loci of circRNAs

Supplementary Table S10: Enrichment Analysis of Significant BMI-SNPs between Females and Males on circRNAs

Supplementary Table S11: Descriptive Statistics of the Normalised Primer Extension Assay Data

Supplementary Figure S1: Significant Enrichment of BMI-SNPs Significant in Females

Supplementary Figure S2: 20 circRNAs with the Highest Numbers of miRNA Target Sites

Supplementary Figure S3: 20 miRNAs with the Highest Numbers of Linked circRNAs

Supplementary Figure S4: Exemplary SNaPshot Output for One Homozygous G/G Carrier of rs4752856

Supplementary Figure S5: Exemplary SNaPshot Output for the Homozygous A/A Carrier of rs4752856

Supplementary Figure S6: Exemplary SNaPshot Output for One Heterozygous (G/A) Carrier of the SNP rs4752856

Supplementary Table S1: Overview of circRNA Datasets.

CircRNA database	Genome version	n circRNAs
circAtlas v2.0[1]	GRCh38	564,515
	GRCh37*	564,456*
circBase[2]	GRCh37	89,374
CIRCpediaV2[3]	GRCh38	179,076
circVAR[4]	GRCh38	286,445

The number of circRNAs reported refers to the total number of circRNAs that remained in the study following the data processing (e.g. removal of inconclusive data). *The circAtlas v2.0 dataset based on the GRCh37 genome version was exclusively used to verify the deviations between BMI-SNPs significant for females and males.

Supplementary Table S2: Overview of Analysed GWAS Data.

GWAS study	Authors	Source	n SNPs	Link to source
**Body mass index**	Yengo et al. 2018	GIANT consortium	2,324,569	https://portals.broadinstitute.org/collaboration/giant/index.php/Main_Page
**Amyotrophic lateral sclerosis (ALS)**	Iacoangeli et al. 2020	NHGRI-EBI GWAS catalog	10,815,403	https://www.ebi.ac.uk/gwas/studies/GCST90013429
**Anorexia nervosa**	Watson et al. 2018	PGC website	8,219,102	https://www.med.unc.edu/pgc/
**Autism spectrum disorder**	Grove et al. 2019	PGC website	9,112,386	https://www.med.unc.edu/pgc/
**Body mass index (female)**	Pulit et al. 2019	GIANT consortium	27,380,251	https://portals.broadinstitute.org/collaboration/giant/index.php/Main_Page
**Body mass index (male)**	Pulit et al. 2019	GIANT consortium	27,380,253	https://portals.broadinstitute.org/collaboration/giant/index.php/Main_Page
**Body height**	Yengo et al. 2018	GIANT consortium website	2,334,001	https://portals.broadinstitute.org/collaboration/giant/index.php/Main_Page
**Chronic Kidney Disease**	Wuttke et al. 2019	NHGRI-EBI GWAS catalog	9,585,588	https://www.ebi.ac.uk/gwas/studies/GCST008064
**Epilepsy**	ILAE consortium et al. 2018	NHGRI-EBI GWAS catalog	4,880,491	https://www.ebi.ac.uk/gwas/studies/GCST007343
**Heart failure**	Shah & Henry et al. 2020	Cardiovascular Disease Knowledge Portal	8,281,262	http://www.broadcvdi.org/
**Pernicious anemia**	Glanville et al. 2021	NHGRI-EBI GWAS catalog	9,419,702	https://www.ebi.ac.uk/gwas/studies/GCST90014450
**Ulcerative colitis**	De Lange et al. 2017	NHGRI-EBI GWAS catalog	9,588,015	https://www.ebi.ac.uk/gwas/studies/GCST004133

The number of SNPs refers to the total number of SNPs included, regardless of the significance level and can deviate from the total set of SNPs included in the respective studies. All data was downloaded in the genome version GRCh37. EBI: European Bioinformatics Institute; GIANT: Genetic Investigation of Anthropometric Traits; GWAS: genome-wide association study; ILAE: International League Against Epilepsy; NHGRI: National Human Genome Research Institute; PGC: Psychiatric Genomics Consortium; SNP: single nucleotide polymorphism.

Supplementary Table S3: Assignment of SNPs of the GWAS Studies Analysing Additional Phenotypes.

Phenotype classification	GWAS	Group classification	Number of SNPs
**Anthropometric**	Body height[5]	Significant ($P < 5*10^{-8}$)	130,933
		Non-significant ($P \geq 5*10^{-8}$)	2,203,068
**Neurological**	Amyotrophic lateral sclerosis[6]	Significant ($P < 5*10^{-8}$)	185
		Non-significant ($P \geq 5*10^{-8}$)	10,815,218
	Epilepsy[7]	Significant ($P < 5*10^{-8}$)	118
		Non-significant ($P \geq 5*10^{-8}$)	4,880,373
**Peripheral**	Chronic kidney disease[8]	Significant ($P < 5*10^{-8}$)	1,443
		Non-significant ($P \geq 5*10^{-8}$)	9,584,145
	Heart failure[9]	Significant ($P < 5*10^{-8}$)	295
		Non-significant ($P \geq 5*10^{-8}$)	8,280,967
	Pernicious anemia[10]	Significant ($P < 5*10^{-8}$)	150
		Non-significant ($P \geq 5*10^{-8}$)	9,419,552
	Ulcerative colitis[11]	Significant ($P < 5*10^{-8}$)	7,697
		Non-significant ($P \geq 5*10^{-8}$)	9,580,318
**Psychiatric**	Anorexia Nervosa[12]	Significant ($P < 5*10^{-8}$)	326
		Non-significant ($P \geq 5*10^{-8}$)	8,218,776
	Autism spectrum disorder[13]	Significant ($P < 5*10^{-8}$)	93
		Non-significant ($P \geq 5*10^{-8}$)	9,112,293

The classification of the SNP data into significant and non-significant variants was based on the genome-wide P-value threshold of $5*10^{-8}$. The sources of the SNP data can be found in Supplementary Table S2.

Supplementary Table S4: List of Used Primers.

Amplicon	Forward primer (5'-3')	Reverse primer (5'-3')
**rs4752856 on gDNA**	AATGGGGTCTTGCTCAGTTG	TCCTGCATGGTTTGGTACTG
**rs4752856 on circRNA (divergent primers)**	AATGGGGTCTTGCTCAGTTG	TCCCTTTGTGCTTGTCTCCA
**rs4752856 SNaPshot- specific primer**	TGTCTGGCCTGAACAATATTATTTC	-
**Digestion control - SHBG**	CTTCCCTGTCTCTCCTCTGGC	CCCAGTGATTGTGCAGTTGG

The primer pair amplifying the SNP rs4752856 on the circRNA was ordered as high performance liquid chromatography (HPLC) purified primers.

Supplementary Table S5: Results of the Analysis of BMI-SNPs Pertaining to their Localisation on circRNA Genomic Loci.

Database	SNP classification	n SNPs on circRNAs (percentage)	n SNPs not on circRNAs (percentage)	Statistics			
				Chi-Square	P-value	OR	95% CI
**circAtlas v2.0**	Significant ($P < 5*10^{-8}$)	25,669 (62.86%)	15,166 (37.14%)	1012	< 0.0001	1.39	[1.36, 1.42]
	Non-significant ($P \geq 5*10^{-8}$)	1,255,157 (54.96%)	1,028,577 (45.04%)				
**circBase**	Significant ($P < 5*10^{-8}$)	9,949 (24.36%)	30,886 (75.64%)	1057	< 0.0001	1.46	[1.43, 1.49]
	Non-significant ($P \geq 5*10^{-8}$)	413,361 (18.10%)	1,870,373 (81.90%)				
**CIRCpediaV2**	Significant ($P < 5*10^{-8}$)	12,913 (31.62%)	27,922 (68.38%)	1721	< 0.0001	1.56	[1.53, 1.59]
	Non-significant ($P \geq 5*10^{-8}$)	522,965 (22.90%)	1,760,769 (77.10%)				
**circVAR**	Significant ($P < 5*10^{-8}$)	15,561 (38.11%)	25,274 (61.89%)	1112	< 0.0001	1.41	[1.38, 1.44]
	Non-significant ($P \geq 5*10^{-8}$)	695,130 (30.44%)	1,588,604 (69.56%)				

The total number and ratio of significant and non-significant SNPs which are either located on circRNAs or outside the genomic loci are shown. The SNPs were extracted from a BMI GWAS meta-analysis performed by Yengo et al. (2018). Results were obtained by applying a custom R script to the SNP and circRNA data of the respective database. The statistical output was generated with GraphPad Prism (version: 9.2.0) using the Chi-square test and the Woolf logit intervals to calculate the 95% confidence interval of the odds ratio. CircAtlas v2.0 results are based on the GRCh38 dataset. OR: odds ratio. P: P-value.

Supplementary Table S6: Results of the Sensitivity Analysis.

Database	SNP classification	n SNPs on circRNAs (ratio)	n SNPs not on circRNAs (ratio)	Statistics			
				Chi-Square	P-value	OR	95% CI
circAtlas v2.0	Significant ($P < 5*10^{-8}$)	25,669 (62.86%)	15,166 (37.14%)	---	---	---	---
	Non-Significant ($P \geq 5*10^{-7}$)	1,246,892 (54.93%)	1,022,903 (45.07%)	1019	< 0.0001	1.39	[1.36, 1.42]
	Non-Significant ($P \geq 5*10^{-6}$)	1,233,915 (54.89%)	1,104,264 (45.11%)	1031	< 0.0001	1.39	[1.36, 1.42]
	Non-Significant ($P \geq 5*10^{-5}$)	1,214,293 (54.82%)	1,000,755 (45.18%)	1047	< 0.0001	1.40	[1.37, 1.42]
circBase	Significant ($P < 5*10^{-8}$)	9,949 (24.36%)	30,886 (75.64%)	---	---	---	---
	Non-Significant ($P \geq 5*10^{-7}$)	410,164 (18.07%)	1,859,631 (81.93%)	1068	< 0.0001	1.46	[1.43, 1.49]
	Non-Significant ($P \geq 5*10^{-6}$)	405,043 (18.02%)	1,843,136 (81.98%)	1089	< 0.0001	1.47	[1.43, 1.50]
	Non-Significant ($P \geq 5*10^{-5}$)	397,889 (17.96%)	1,817,159 (82.04%)	1109	< 0.0001	1.47	[1.44, 1.51]
CIRCpediaV2	Significant ($P < 5*10^{-8}$)	12,913 (31.62%)	27,922 (68.38%)	---	---	---	---
	Non-Significant ($P \geq 5*10^{-7}$)	519,175 (22.87%)	1,750,620 (77.13%)	1732	< 0.0001	1.56	[1.53, 1.59]
	Non-Significant ($P \geq 5*10^{-6}$)	512,978 (22.82%)	1,735,201 (77.18%)	1757	< 0.0001	1.56	[1.53, 1.60]
	Non-Significant ($P \geq 5*10^{-5}$)	504,181 (22.76%)	1,710,867 (77.24%)	1782	< 0.0001	1.57	[1.54, 1.60]
circVAR	Significant ($P < 5*10^{-8}$)	15,561 (38.11%)	25,274 (61.89%)	---	---	---	---
	Non-Significant ($P \geq 5*10^{-7}$)	690,154 (30.41%)	1,579,641 (69.59%)	1121	< 0.0001	1.41	[1.38, 1.44]
	Non-Significant ($P \geq 5*10^{-6}$)	682,152 (30.34%)	1,566,027 (69.66%)	1141	< 0.0001	1.41	[1.39, 1.44]
	Non-Significant ($P \geq 5*10^{-5}$)	670,786 (30.28%)	1,544,262 (69.72%)	1159	< 0.0001	1.42	[1.39, 1.45]

Our sensitivity analysis led to a more distinct delimitation of significant and non-significant SNPs by defining three novel P-value cut-offs and removing all non-significant SNPs with a P-value within the range of these novel thresholds and the initial one ($5*10^{-8}$). The SNPs were extracted from the BMI GWAS meta-analysis performed by Yengo et al. (2018). Results were obtained by applying a custom R script to the re-defined SNP data and the circRNA data of the respective databases. The statistical output was generated with GraphPad Prism (version 9.2.0) using a two-sided Chi-square test and the Woolf logit intervals to calculate the 95% confidence intervals of the odds ratio. Results of the circAtlas v2.0 dataset are based on the GRCh38-derived data. CI: confidence interval. OR: odds ratio.

Supplementary Table S7: Results When Considering the Linkage Disequilibrium of SNPs.

Database	SNP classification	n SNPs on circRNAs (ratio)	n SNPs not on circRNAs (ratio)	Statistics			
				Chi-square	P-value	OR	95% CI
circAtlas v2.0	Significant (within 1 Mb region)	457,487 (56.77%)	348,421 (43.23%)	1386	< 0.0001	1.11	[1.10, 1.12]
	Non-significant (not within 1 Mb region)	823,339 (54.21%)	695,322 (45.79%)				
	Significant (in regions of high LD)	45,166 (58.58%)	31,938 (41.42%)	390	< 0.0001	1.16	[1.14, 1.18]
	Non-significant (not within regions of high LD)	1,235,660 (54.50%)	1,011,805 (45.50%)				
circBase	Significant (within 1 Mb region)	161,311 (20.02%)	644,597 (79.98%)	2701	< 0.0001	1.20	[1.19, 1.21]
	Non-significant (not within 1 Mb region)	261,999 (17.25%)	1,256,662 (82.75%)				
	Significant (in regions of high LD)	16,588 (21.51%)	60,516 (78.49%)	584.3	< 0.0001	1.24	[1.22, 1.26]
	Non-significant (not within regions of high LD)	406,722 (18.10%)	1,840,743 (81.90%)				
CIRCpediaV2	Significant (within 1 Mb region)	205,663 (25.52%)	600,245 (74.48%)	4231	< 0.0001	1.23	[1.23, 1.24]
	Non-significant (not within 1 Mb region)	330,215 (21.74%)	1,188,446 (78.26%)				
	Significant (in regions of high LD)	21,030 (27.27%)	56,074 (72.73%)	801.4	< 0.0001	1.26	[1.24, 1.28]
	Non-significant (not within regions of high LD)	514,848 (22.91%)	1,732,617 (77.09%)				
circVAR	Significant (within 1 Mb region)	262,084 (32.52%)	543,824 (67.48%)	2204	< 0.0001	1.15	[1.14, 1.16]
	Non-significant (not within 1 Mb region)	448,607 (29.54%)	1,070,054 (70.46%)				
	Significant (in regions of high LD)	26,314 (34.13%)	50,790 (65.87%)	474.8	< 0.0001	1.18	[1.17, 1.20]
	Non-significant (not within regions of high LD)	684,377 (30.45%)	1,563,088 (69.55%)				

To correct for the linkage disequilibrium of the SNPs, we re-defined all non-significant SNPs within a 1 Mb region surrounding a significant SNP ($P < 5*10^{-8}$) and non-significant SNPs within regions of high LD (as stated in plinkQC[14]) as significant as well. The data displayed is based on the SNP data extracted from the BMI GWAS meta-analysis conducted by Yengo et al. (2018). The two-sided Chi-square test and odds ratio using the Woolf logit intervals were calculated with GraphPad Prism (version 9.2.0). The significant level was set to 95%. Results of the circAtlas v2.0 dataset are based on the GRCh38 data. CI: confidence interval. LD: linkage disequilibrium. OR: odds ratio.

Supplementary Table S8: CircRNA Location of SNPs Extracted from GWAS Pertaining Various Phenotypes.

Phenotype classification	GWAS	SNP classification	n SNPs on circRNAs (ratio)	n SNPs not on circRNAs (ratio)	Statistics			
					Chi-Square	P-value*	OR	95% CI
**Anthropometric**	Body height[5]	Significant	41,529 (31.72%)	89,404 (68.28%)	16,787	< 0.0001	2.19	[2.17, 2.22]
		Non-significant	384,987 (17.48%)	1,818,081 (82.52%)				
**Neurological**	Amyotrophic lateral sclerosis[6]	Significant	18 (9.73%)	167 (90.27%)	9.08	0.003	0.48	[0.30, 0.78]
		Non-significant	1,978,337 (18.29%)	8,836,881 (81.71%)				
	Epilepsy[7]	Significant	10 (8.47%)	108 (91.53%)	7.05	0.008	0.43	[0.22, 0.82]
		Non-significant	870,094 (17.83%)	4,010,279 (82.17%)				
**Peripheral**	Chronic kidney disease[8]	Significant	450 (31.19%)	993 (68.81%)	162.7	<0.0001	2.03	[1.82, 2.27]
		Non-significant	1,746,337 (18.22%)	7,837,808 (81.78%)				
	Heart failure[9]	Significant	59 (20%)	236 (80%)	0.78	0.38	1.14	[0.85, 1.51]
		Non-significant	1,492,677 (18.03%)	6,788,290 (81.97%)				
	Pernicious anemia[10]	Significant	13 (8.67%)	137 (91.33%)	8.99	0.003	0.43	[0.24, 0.76]
		Non-significant	1,704,296 (18.09%)	7,715,256 (81.91%)				
	Ulcerative colitis[11]	Significant	1,096 (14.24%)	6,601 (85.76%)	88.75	< 0.0001	0.74	[0.69, 0.78]
		Non-significant	1,762,928 (18.40%)	7,817,390 (81.60%)				
**Psychiatric**	Anorexia nervosa[12]	Significant	168 (51.53%)	158 (48.47%)	244.7	< 0.0001	4.83	[3.88, 6.00]
		Non-significant	1,483,926 (18.06%)	6,734,850 (81.94%)				
	Autism spectrum disorder[13]	Significant	40 (43.01%)	53 (56.99%)	36.80	< 0.0001	3.40	[2.26, 5.12]
		Non-significant	1,655,688 (18.17%)	7,456,605 (81.83%)				

To explore whether the detected enrichment of BMI-associated SNPs on circRNA genomic loci can be detected in further phenotypes and traits, we analysed additional GWAS studies pertaining anthropometric traits, neurological, peripheral and psychiatric disorders. The SNPs were classified based on their P-value into sets of significant ($P < 5*10^{-8}$) and non-significant ($P \geq 5*10^{-8}$) variants. The custom R script was applied for those SNPs and the circRNA data extracted exclusively from circBase. The two-sided Chi-square test and the odds ratio applying the Woolf logit intervals were computed with GraphPad Prism (version 9.2.0). The significance level was set to 95%. The Bonferroni correction was performed based on the total number of GWAS studies investigated (n = 10; as stated in Table 1). Thus, the corrected P-value threshold is 0.005 (*). CI: confidence interval. OR: odds ratio.

Supplementary Table S9: Gender-Separated Analysis of BMI-SNPs on the Genomic Loci of circRNAs.

GWAS	Gender	SNP classification	Number of SNPs on circRNAs (ratio)	Number of SNPs not on circRNAs (ratio)	Statistics			
					Chi-Square	P-value	OR	95% CI
**BMI (Pulit et al. 2019)**	Female	Significant ($P < 5*10^{-8}$)	7,109 (25.71%)	20,544 (74.29%)	715.5	< 0.0001	1.44	[1.40,1.48]
		Non-significant ($P \geq 5*10^{-8}$)	5,292,550 (19.35%)	22,060,048 (80.65%)				
	Male	Significant ($P < 5*10^{-8}$)	5,372 (23.52%)	17,461 (76.48%)	254.8	< 0.0001	1.28	[1.24, 1.32]
		Non-significant ($P \geq 5*10^{-8}$)	5,294,287 (19,35%)	22,063,133 (80.65%)				

An additional BMI GWAS[15] was analysed to investigate whether deviations of circRNA genomic loci localisation of genome-wide significant BMI-SNPs between males and females are present. Therefore, the respective SNP data was grouped based on the P-value of $5*10^{-8}$. The two-sided Chi-square test and the odds ratio based on the Woolf logit intervals (confidence level: 95%) were computed with GraphPad Prism (version 9.2.0). CI: confidence interval. OR: odds ratio.

Supplementary Table S10: Enrichment Analysis of Significant BMI-SNPs between Females and Males on circRNAs.

GWAS	Database	Gender	SNP classification	Number of SNPs on circRNAs (ratio)	Number of SNPs not on circRNAs (ratio)	Statistics			
						Chi-Square	P-value	OR	95% CI
**BMI (Pulit et al. 2019)**	circBase (hg19)	Female	Significant ($P < 5*10^{-8}$)	7,109 (25.71%)	20,544 (74.29%)	31.95	< 0.0001	1.13	[1.08, 1.17]
		Male	Significant ($P < 5*10^{-8}$)	5,372 (23.52%)	17,461 (76.48%)				
	circAtlas v2.0 (hg19)	Female	Significant ($P < 5*10^{-8}$)	17,439 (63.06%)	10,214 (36.94%)	18.98	< 0.0001	1.08	[1.05, 1.12]
		Male	Significant ($P < 5*10^{-8}$)	13,968 (61.17%)	8,865 (38.83%)				

It was analysed whether deviations of circRNA-located and significant BMI-SNPs between females and males are present. Therefore, significant SNPs ($P < 5*10^{-8}$) in females and males extracted from a gender-specific GWAS for BMI[15] were investigated. For the analysis of the circAtlas v2.0 data, an additional dataset based on the older genome version, GRCh37, was downloaded from the circAtlas v2.0 website. This contained marginally less (564,456 circRNAs in total) than the GRCh38 dataset (see Supplementary Table S1). The two-sided Chi-square test and the odds ratio based on the Woolf logit intervals (confidence level 95%) were calculated with GraphPad Prism (version 9.2.0). CI: confidence interval. OR: odds ratio.

Supplementary Table S11: Descriptive Statistics of the Normalised Primer Extension Assay Data.

Subject	Mean	Standard error	95% CI	Median	Variance	Standard deviation
**Subject 1**	1.17	0.08	[0.82, 1.52]	1.18	0.02	0.14
**Subject 2**	1.21	0.11	[0.74, 1.68]	1.17	0.04	0.19
**Subject 3**	1.34	0.08	[1.01, 1.67]	1.41	0.02	0.13
**Subject 6**	1.30	0.11	[0.81, 1.79]	1.33	0.04	0.20
**Subject 7**	1.29	0.03	[1.17, 1.40]	1.29	0.002	0.05
**Subject 8**	1.22	0.07	[0.92, 1.51]	1.28	0.01	0.12
**Subject 9**	1.22	0.07	[0.94, 1.51]	1.19	0.01	0.11
**Subject 10**	2.16	0.17	[1.41, 2.91]	2.19	0.09	0.30
**All**	1.36	0.12	[1.09, 1.64]	1.255	0.11	0.33

The data is based on the A/G circRNA/DNA normalised data produced in the primer extension assay (see Equation (1) and see Table 2). The descriptive statistics were calculated with SPSS. The confidence intervals were computed based on the t-test.

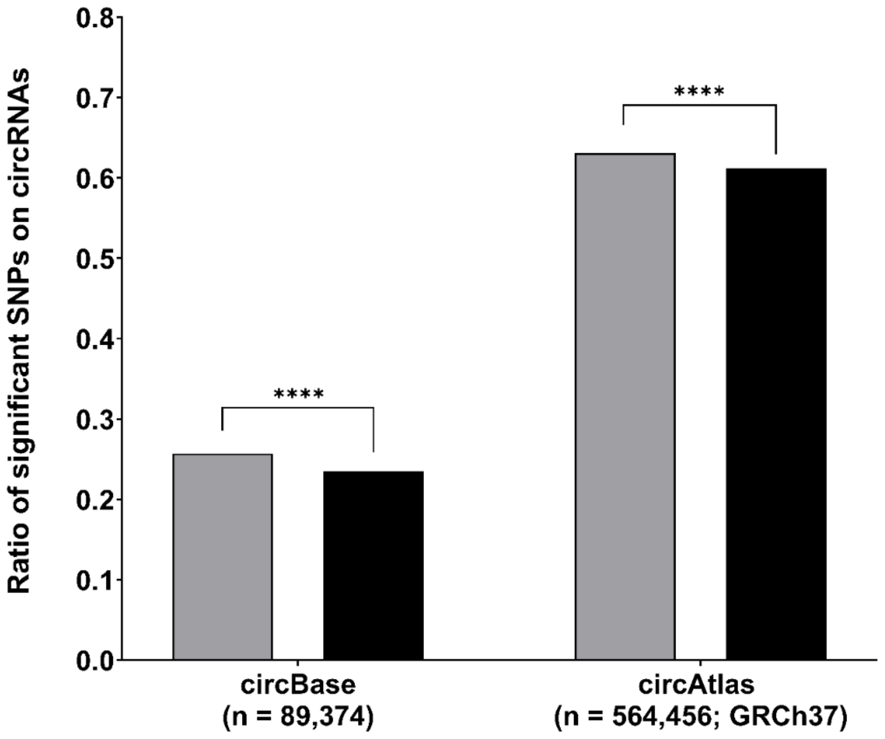

Supplementary Figure S1: Significant Enrichment of BMI-SNPs Significant in Females. Here, SNPs classified as significant ($P < 5*10^{-8}$) in females (grey) and males (black) were analysed regarding their circRNA localisation using a custom R script. The numbers shown in the parentheses represent the number of circRNAs included in the corresponding dataset. For this analysis, an alternative circAtlas v2.0 dataset based on the older genome version GRCh37 was used. This contained marginally less circRNAs than the GRCh38 dataset (see Supplementary Table S1). The results of the statistical test can be found in Supplementary Table S10. **** $P < 0.0001$.

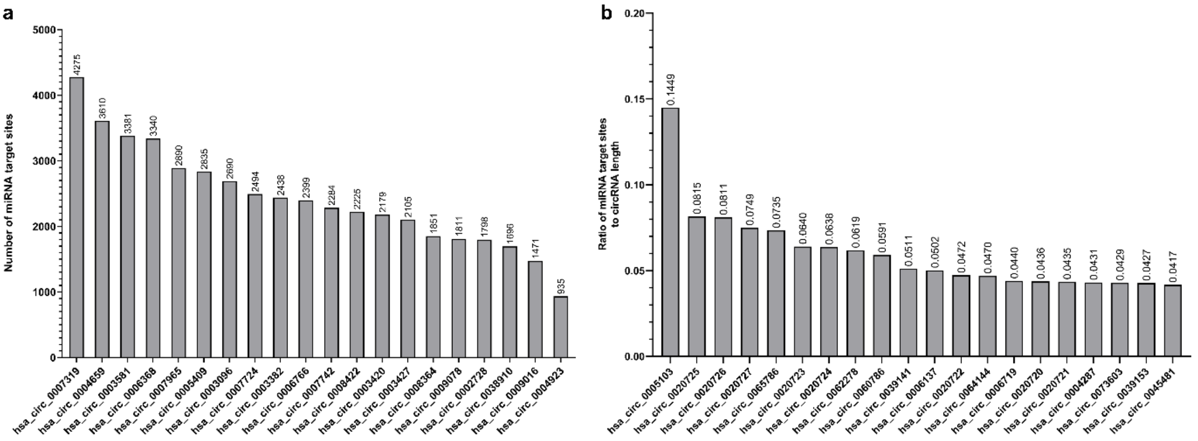

Supplementary Figure S2: 20 circRNAs with the Highest Numbers of miRNA Target Sites. Correlations of miRNAs and circRNAs were investigated for circBase-derived circRNAs containing at least one significant SNP for BMI ($P < 5*10^{-8}$; 9,949 SNPs; see Supplementary Table S5). An additional dataset from the Encyclopedia of RNA Interactions (ENCORI[16]) database was downloaded. This included data regarding already known miRNA-circRNA interactions of circBase circRNAs supported by Ago CLIP-sequencing data. Here, the 20 circRNAs with the highest raw numbers of miRNAs linked (a) and the highest ratio of miRNAs normalised on the total length of the circRNA (b) are represented. The length of the circRNAs was calculated with the start and stop position stated in the circBase dataset.

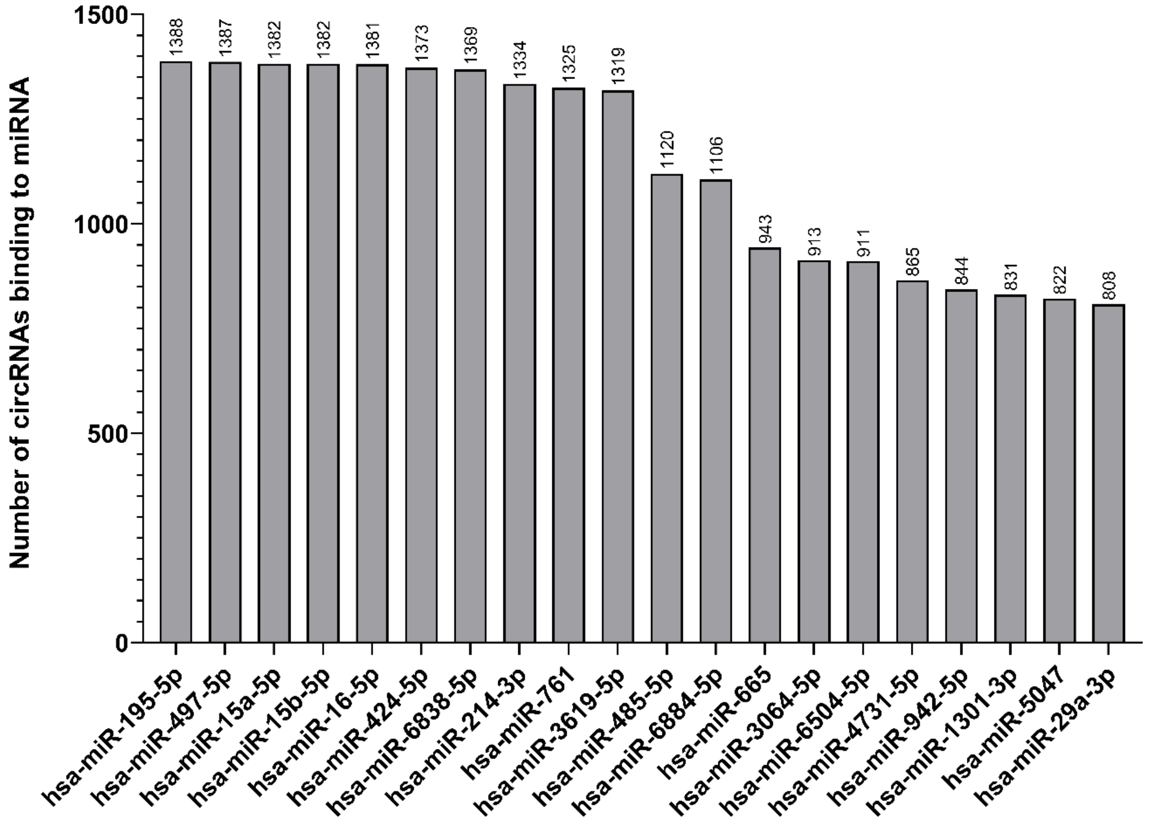

Supplementary Figure S3: 20 miRNAs with the Highest Numbers of Linked circRNAs. To explore putative miRNA-circRNA interactions, a dataset containing known interactions supported by Ago CLIP-sequencing data was downloaded from the ENCORI[16]. Here, the 20 miRNAs with the highest raw numbers of circRNAs linked to are represented.

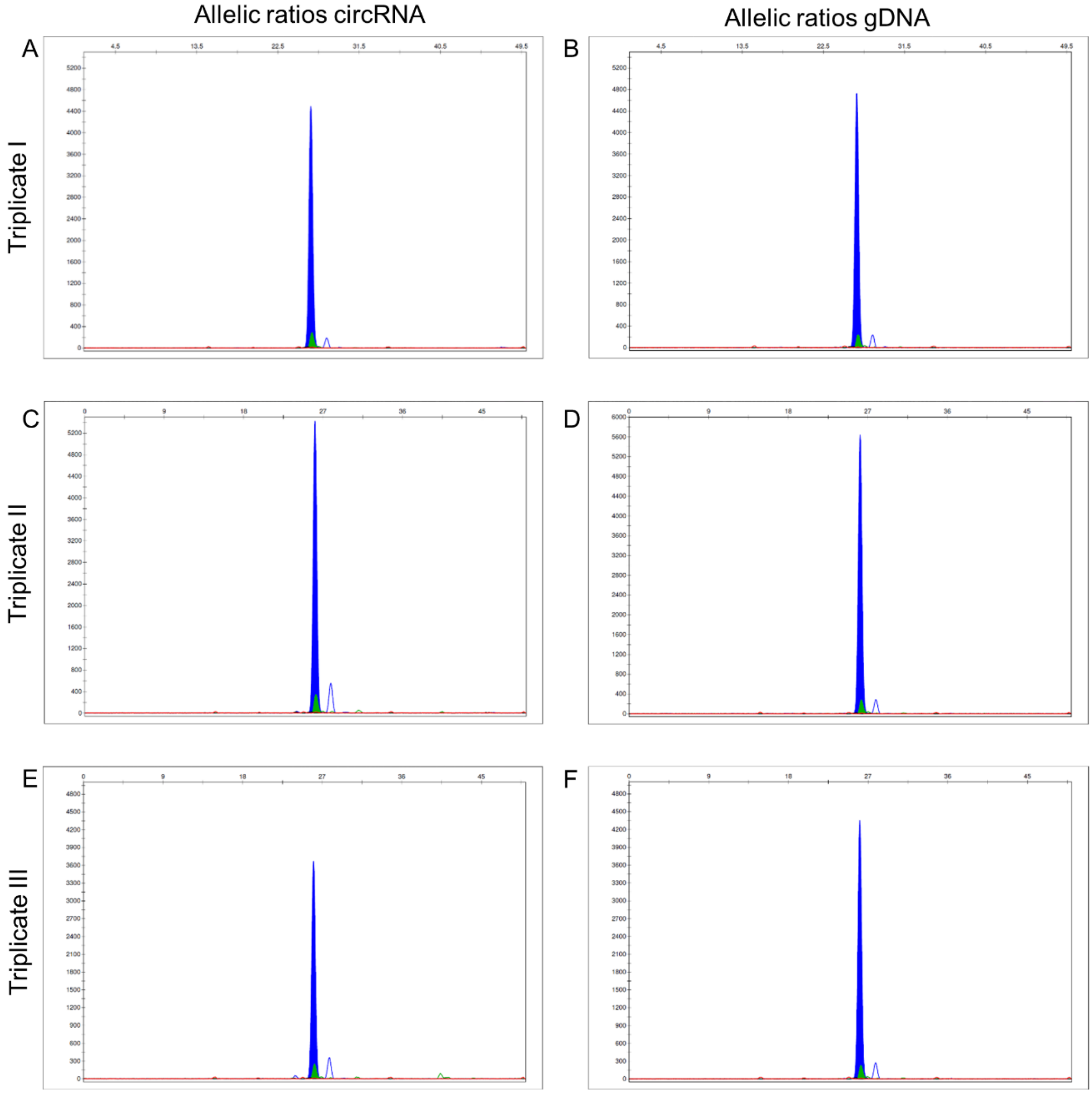

Supplementary Figure S4: Exemplary SNaPshot Output for One Homozygous G/G Carrier of rs4752856. Here, the primer extension products containing the G-allele of the circRNA (A, C and E) and of the genomic DNA (B, D and F) are shown for all three triplicates. As homozygous individuals solely acted as an experimental controls, the yielded data was not further analysed. These plots were generated with the GeneMapper 4.0 software (Applied Biosystems, Foster City, CA, USA). Blue: G-allele

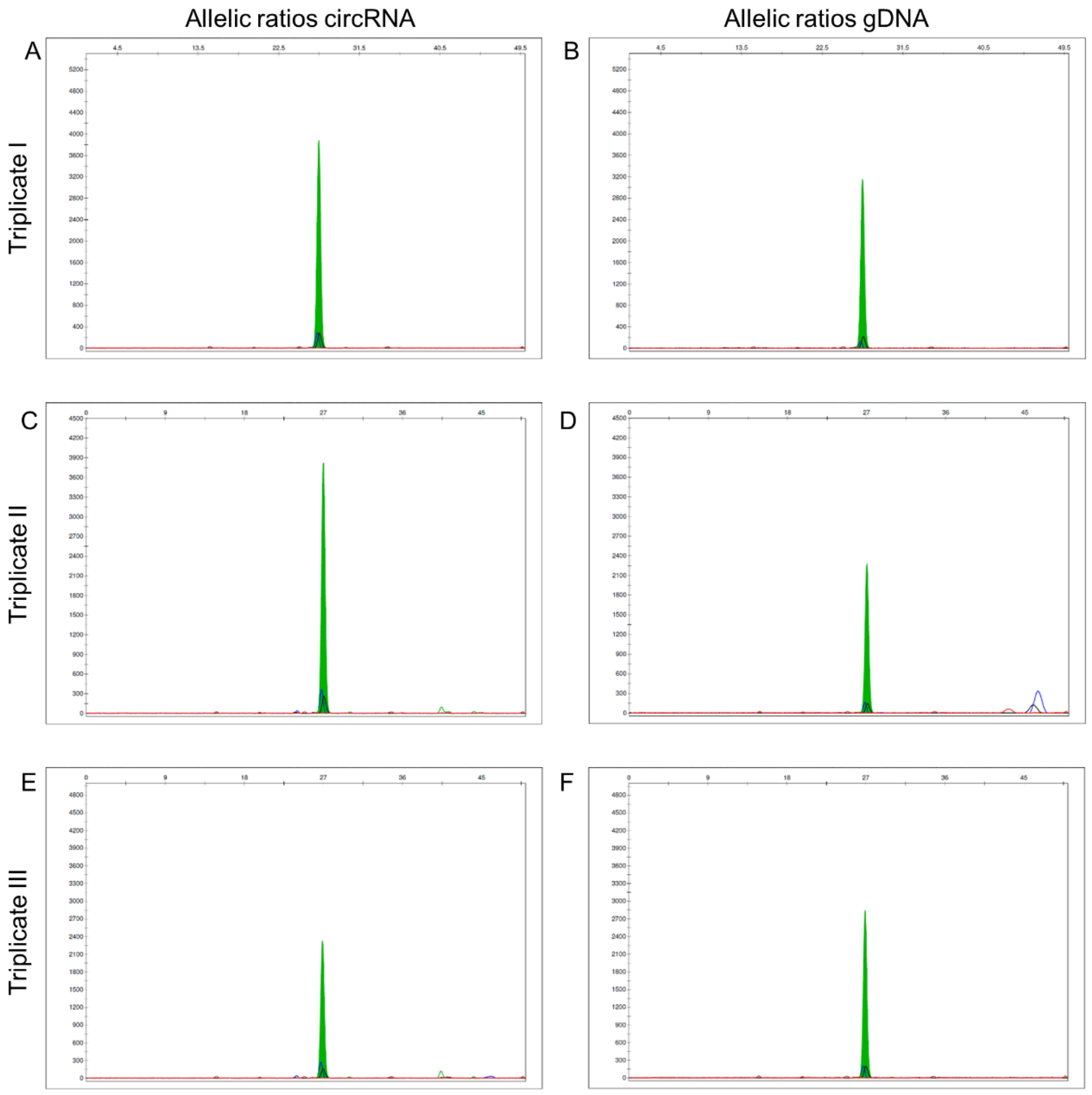

Supplementary Figure S5: Exemplary SNaPshot Output for the Homozygous A/A Carrier of rs4752856. Here, the primer extension products containing the A-allele of the circRNA (A, C and E) and genomic DNA (B, D and F) are shown. The experiment was performed in triplicates. As this homozygous individual acted as an experimental internal control, no further analyses were conducted. Green: A-allele (risk allele).

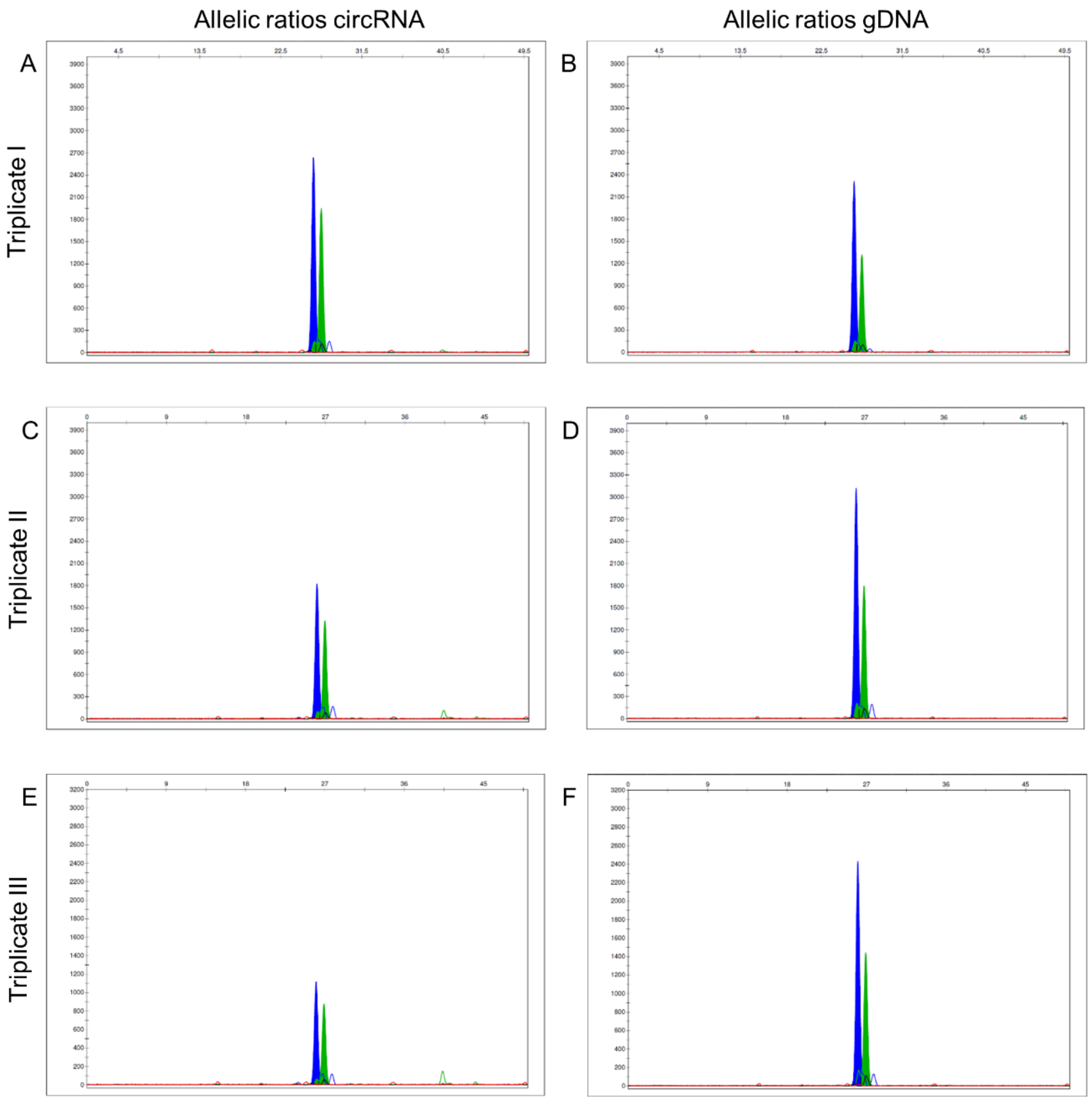

Supplementary Figure S6: Exemplary SNaPshot Output for One Heterozygous (G/A) Carrier of the SNP rs4752856. The primer extension products of a heterozygous carrier (here: subject 7) as presented in the GeneMapper 4.0 software (Applied Biosystems, Foster City, CA, USA) on the circRNA (A, C and E) and on the gDNA (B, D and F) are shown. This assay was performed in triplicates. The corresponding analysis of the allelic ratios (A/G) as well as the normalisation of the ratios of circRNA with genomic DNA are represented in Figure 6, Table 2 and Supplementary Table S11. Blue: G-allele. Green: A-allele (risk allele).

References:

1. Wu, W., Ji, P. & Zhao, F. CircAtlas: an integrated resource of one million highly accurate circular RNAs from 1070 vertebrate transcriptomes. *Genome Biol* **21**, 101 (2020).
2. Glažar, P., Papavasileiou, P. & Rajewsky, N. circBase: a database for circular RNAs. *RNA* **20**, 1666-70 (2014).
3. Dong, R., Ma, X.K., Li, G.W. & Yang, L. CIRCpedia v2: An Updated Database for Comprehensive Circular RNA Annotation and Expression Comparison. *Genomics Proteomics Bioinformatics* **16**, 226-233 (2018).
4. Zhao, M. & Qu, H. circVAR database: genome-wide archive of genetic variants for human circular RNAs. *BMC Genomics* **21**, 750 (2020).
5. Yengo, L. *et al.* Meta-analysis of genome-wide association studies for height and body mass index in approximately 700000 individuals of European ancestry. *Hum Mol Genet* **27**, 3641-3649 (2018).
6. Iacoangeli, A. *et al.* Genome-wide Meta-analysis Finds the ACSL5-ZDHHC6 Locus Is Associated with ALS and Links Weight Loss to the Disease Genetics. *Cell Rep* **33**, 108323 (2020).
7. International League Against Epilepsy Consortium on Complex, E. Genome-wide mega-analysis identifies 16 loci and highlights diverse biological mechanisms in the common epilepsies. *Nat Commun* **9**, 5269 (2018).
8. Wuttke, M. *et al.* A catalog of genetic loci associated with kidney function from analyses of a million individuals. *Nat Genet* **51**, 957-972 (2019).
9. Shah, S. *et al.* Genome-wide association and Mendelian randomisation analysis provide insights into the pathogenesis of heart failure. *Nat Commun* **11**, 163 (2020).
10. Glanville, K.P., Coleman, J.R.I., O'Reilly, P.F., Galloway, J. & Lewis, C.M. Investigating Pleiotropy Between Depression and Autoimmune Diseases Using the UK Biobank. *Biol Psychiatry Glob Open Sci* **1**, 48-58 (2021).
11. de Lange, K.M. *et al.* Genome-wide association study implicates immune activation of multiple integrin genes in inflammatory bowel disease. *Nat Genet* **49**, 256-261 (2017).
12. Watson, H.J. *et al.* Genome-wide association study identifies eight risk loci and implicates metabo-psychiatric origins for anorexia nervosa. *Nat Genet* **51**, 1207-1214 (2019).
13. Grove, J. *et al.* Identification of common genetic risk variants for autism spectrum disorder. *Nat Genet* **51**, 431-444 (2019).
14. Meyer, H.V. plinkQC: Genotype quality control in genetic association studies. (2020).
15. Pulit, S.L. *et al.* Meta-analysis of genome-wide association studies for body fat distribution in 694 649 individuals of European ancestry. *Hum Mol Genet* **28**, 166-174 (2019).
16. Li, J.H., Liu, S., Zhou, H., Qu, L.H. & Yang, J.H. starBase v2.0: decoding miRNA-ceRNA, miRNA-ncRNA and protein-RNA interaction networks from large-scale CLIP-Seq data. *Nucleic Acids Res* **42**, D92-7 (2014).
